# Acute Presentations of Colorectal Cancer: An International Prospective Snapshot on Management and Outcomes

**DOI:** 10.1002/wjs.70106

**Published:** 2025-10-28

**Authors:** EuroSurg Collaborative, STARSurg Collaborative

**Keywords:** APOLLO, bowel obstruction, bowel perforation, colorectal cancer, emergency surgery, global surgery

## Abstract

**Background:**

Patients with colorectal cancer (CRC) present as an emergency in one‐third of cases. This international snapshot study describes the presentation, treatment, and overall outcomes of patients admitted acutely to hospital with CRC.

**Methods:**

Acute presentation of colorectal cancer—an international snapshot (APOLLO) was a prospective, international, cohort study enrolling consecutive patients admitted acutely to hospital with CRC from January to June 2023. The primary outcome was mortality at 90 days. Reasons for acute CRC presentation are described, as are the rates of surgical and nonsurgical management and mortality.

**Results:**

Overall, 1861 patients (257 centers, 39 countries) were included (median age 70 years; females 44.8%). Rates of obstruction (54.5%; 1015 of 1861) and perforation (11.4%; 212 of 1861) are quantified. In total, 59.2% of patients were managed with curative intent (1102 of 1861). Curative management of right colon cancer predominantly involved resection and primary anastomosis (RPA) (78.5%). Curative management of left colon cancer predominantly involved either RPA or resection with end stoma formation (RES) (42.9% vs. 40.6%). Acute curative rectal cancer management strategies were more heterogeneous (RPA 21.8%, RES 26.7%, diverting stoma only 15.8%, no surgery 31.5%). Overall 90‐day mortality was 17.5% (326 of 1861) and lower in those undergoing curative intent surgery (69 [7.3%] of 949).

**Conclusion:**

This international prospective snapshot study found a 17.5% 90‐day mortality in patients presenting acutely with CRC, predominantly for obstruction. High levels of variation in the management of acutely presenting left colon cancer and rectal cancer are highlighted.

## Graphical abstract

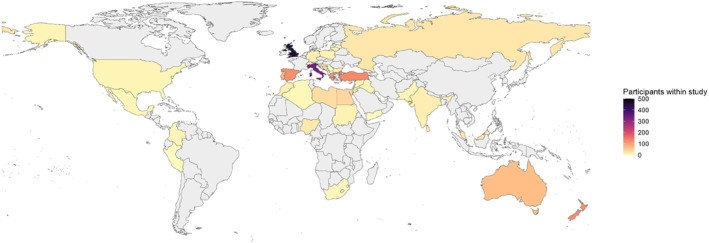

## 1 Introduction

Colorectal cancer (CRC) remains a common disease worldwide, accounting for 10% of all cancers, and is the third leading cause of cancer‐related mortality [1]. Despite advances in cancer screening and treatment efficacy, CRC remains a significant global health concern, with a rising proportion of patients diagnosed at a younger age, an increasing proportion of patients diagnosed at advanced disease stages [2], and little change in long‐term survival rates over the past several decades [3].

CRC presents as an emergency in as many as a third of patients, most significantly as bowel obstruction or perforation [4]. Current management guidelines on CRC emergencies are largely based on consensus statements or data from observational studies of limited size [5, 6]. The lack of high‐quality global data in this area limits our understanding of the presentation, initial management, and outcomes of CRC emergencies. Insights into current trends, including frequency of obstruction and perforation in an era of CRC screening programs, rates of nonoperative management, and the prevalence of colonic stenting, are needed to capture current practice.

To address this issue, we designed the acute presentation of colorectal cancer (APOLLO) study as a global, prospective, snapshot study. This planned secondary analysis of the APOLLO study aimed to (a) characterize the reasons for acute CRC hospital admission, (b) describe the different treatment strategies employed, and (c) define the short‐term outcomes of mortality and complications.

## 2 Methods

### 2.1 Study Design

APOLLO was a prospective, international, multicenter, cohort study, delivered by the student‐ and trainee‐led EuroSurg Collaborative and STARSurg Collaborative, and conducted in accordance to a study protocol published a priori [7]. In the United Kingdom, Caldicott guardian approvals were obtained before recruitment and the study was registered as a service evaluation since no changes to clinical care were made. Elsewhere, local approval was obtained at each participating site in line with local protocols. As this was an observational study without direct interaction with patients, a waiver of consent was obtained for most centers, with requirements for individual consent modified on an individual center as‐needed basis. Collaborators were required to complete a mandatory data governance e‐learning module prior to initiation of data collection. This study was conducted in line with the STROBE reporting guidelines for observational studies [8].

### 2.2 Inclusion Criteria

Consecutive adult patients (> 18 years) presenting acutely (i.e., unplanned and nonelective presentation to hospital for urgent or emergency reasons) and requiring admission for symptoms of previously or newly diagnosed colorectal adenocarcinoma were included regardless of surgical management or disease extent. Patients managed with both attempted curative and palliative intent were included. Patients with previously diagnosed CRC with symptoms related to the disease or tumor progression were also included.

Patients were excluded if they presented acutely for the side effects of chemotherapy or radiotherapy. Patients not requiring admission, with benign or malignant histology different from adenocarcinoma, or patients with noncolorectal primary cancers that metastasize to the colon or rectum (e.g., melanoma or lymphoma) were excluded.

### 2.3 Data Collection

Collaborators from participating centers identified all consecutive eligible patients within data inclusion periods of any consecutive 6‐week period between January and June 2023. The study was open to any secondary or tertiary hospital across the world with a general or colorectal surgery department performing major colorectal surgery.

Data were collected on patient demographics, country income status as defined by the Organization for Economic Co‐operation and Development (OECD) [9], comorbidities defined by the American Society of Anesthesiologists (ASA) Physical Status Grade [10], Clinical Frailty Score [11], cancer location, TNM stage, and curative or palliative treatment intent. Reasons for acute presentation were collected and stratified as bowel obstruction, bowel perforation, and other reasons (including gastrointestinal bleeding and abdominal pain among other reasons—for a comprehensive list of presentations included within other reasons, please refer to the protocol).

CRC location was determined by anatomical location. Tumors of the cecum, ascending colon, and hepatic flexure were classified as right‐sided colon cancers. If a cancer involved the transverse colon but not the splenic flexure, then it was also classified as a right‐sided cancer. Tumors involving the splenic flexure, descending, and sigmoid colon and sparing the rectum were classified as left‐sided colon cancers. If the tumor involved the rectum, with or without the sigmoid colon, then it was classified as a rectal cancer. If cancers involved more than one section of nonadjacent bowel, they were classified as synchronous tumors.

Patient management strategies were stratified: (a) based on whether the cancer was newly diagnosed on this admission (index admission) or previously diagnosed, (b) by cancer location, and (c) by the presence of bowel obstruction or perforation. Management strategies were broadly categorized into nonsurgical management; formation of a diverting stoma without resection; resection with end stoma formation; resection with primary anastomosis with or without the formation of a defunctioning stoma; and laparotomy/laparoscopy without resection or stoma formation. Patients in the nonsurgical management group included patients presenting acutely without a surgical emergency, subsequently discharged with a curative plan, and those presenting with a surgical emergency who were palliated due to being unfit for surgery. Treatment intent was determined from clinical records including hospital records and multidisciplinary meeting outcomes.

Data validation was performed at the end of data collection at each center to verify the case ascertainment rate over the study period. If additional cases meeting inclusion criteria were identified, data were entered subsequently. If during validation cases were identified as not meeting inclusion criteria, they were excluded. For the remaining records, data collection accuracy was cross verified against eight prespecified variables (age, sex, ASA grade, clinical frailty score, reason for presentation, management, length of stay, and Clavien–Dindo Grade).

### 2.4 Outcomes

The primary outcome was mortality at 90 days. Secondary outcomes were mortality at 30 day, rates of primary anastomosis versus stoma formation, rates of nonoperative management, and rates of colonic stenting.

### 2.5 Statistical Analysis

Analyses were completed in R for statistical computing (Vienna, Austria. R Core Team). Differences between categories were tested using *χ*2 tests for categorical variables and Mann–Whitney U/Kruskal–Wallis tests for continuous variables. Data were stratified via newly diagnosed (index) and previously diagnosed CRC.

## 3 Results

**FIGURE 1:**
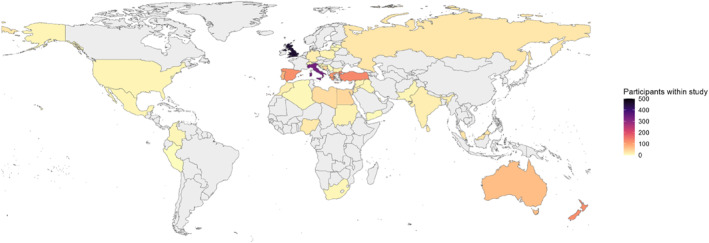
Participant contribution by country.

**FIGURE 2:**
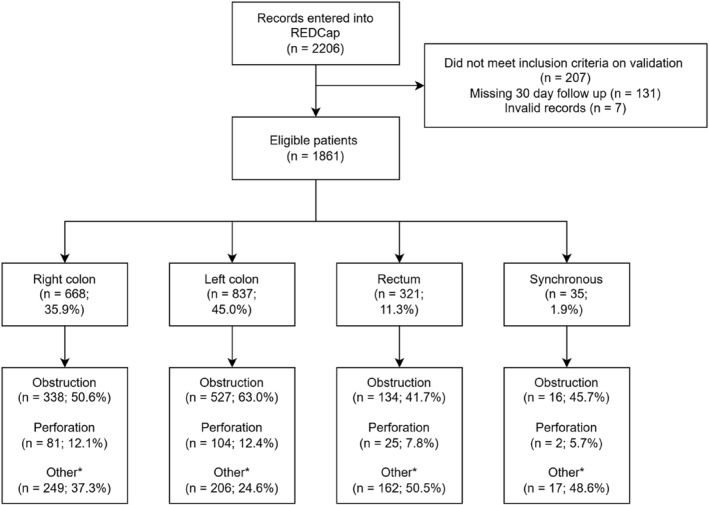
Overview of patient inclusion and reasons for presentation. Patient inclusion flow diagram. Other reasons for presentation listed in Supporting Information S1: table 2.

The APOLLO study recruited 1861 patients (median age 70.0 years; IQR, 58–79, 44.8% female) from 257 centers in 39 countries (median 6 patients per hospital, IQR 3–9, Figure 1 **,** Supporting Information S1: Table 1). Patient exclusions, cancer location, and presenting symptoms are represented by the flowchart in Figure 2.

Among patients acutely presenting to the hospital with CRC, most presented with bowel obstruction (1015 [54.5%] of 1861). Bowel perforation occurred in a smaller proportion (212 [11.4%] of 1861, Figure 2, Table 1). Gastrointestinal (GI) bleeding also commonly occurred (386 [20.7%] of 1861), with the remaining reasons for presentation summarized in Supporting Information S1: Table 2.

**TABLE 1:** Demographics of included participants.

Variable		Curative	Palliative	Total	*p*
Total N		1102	756	1861	
Age	Median (IQR)	69.0 (58.0–77.0)	71.0 (60.0–82.0)	70.0 (58.0–79.0)	< 0.001
Sex	Female	485 (44.0)	346 (45.8)	834 (44.8)	0.484
BMI	< 18.5	58 (5.3)	63 (8.3)	121 (6.5)	0.014
	18.5–24.9	464 (42.1)	296 (39.2)	760 (40.9)	
	25–29.9	328 (29.8)	181 (23.9)	509 (27.4)	
	30–39.9	144 (13.1)	106 (14.0)	250 (13.5)	
	> 40	21 (1.9)	16 (2.1)	37 (2.0)	
	(Missing)	87 (7.9)	94 (12.4)	181 (9.7)	
ASA	I–II	618 (56.1)	298 (39.4)	918 (49.3)	< 0.001
	III–V	444 (40.3)	395 (52.2)	839 (45.1)	
	Not recorded	40 (3.6)	63 (8.3)	104 (5.6)	
Clinical frailty score (1–9)	Median (IQR)	3.0 (2.0–4.0)	4.0 (3.0–6.0)	3.0 (2.0–5.0)	< 0.001
Smoking status	Current	168 (15.2)	99 (13.1)	268 (14.4)	0.026
	Ex‐smoker	222 (20.1)	160 (21.2)	383 (20.6)	
	Never smoked	499 (45.3)	311 (41.1)	810 (43.5)	
	Unknown	213 (19.3)	186 (24.6)	400 (21.5)	
Previous abdominal surgery	No	700 (63.5)	445 (58.9)	1147 (61.6)	0.045
	Yes	401 (36.4)	311 (41.1)	713 (38.3)	
	(Missing)	1 (0.1)	0 (0.0)	1 (0.1)	
Presenting reason	Obstruction	578 (52.5)	437 (57.8)	1015 (54.5)	0.006
	Perforation	118 (10.7)	94 (12.4)	212 (11.4)	
	Other	406 (36.8)	225 (29.8)	634 (34.1)	
Tumor location	Right	395 (35.8)	271 (35.8)	668 (35.9)	< 0.001
	Left	525 (47.6)	311 (41.1)	837 (45.0)	
	Rectum	165 (15.0)	156 (20.6)	321 (17.2)	
	Synchronous	17 (1.5)	18 (2.4)	35 (1.9)	
T stage	T1	101 (9.2)	31 (4.1)	133 (7.1)	< 0.001
	T2	160 (14.5)	54 (7.1)	215 (11.6)	
	T3	490 (44.5)	244 (32.3)	735 (39.5)	
	T4	344 (31.2)	410 (54.2)	754 (40.5)	
	(Missing)	7 (0.6)	17 (2.2)	24 (1.3)	
N stage	N0	401 (36.4)	123 (16.3)	525 (28.2)	< 0.001
	N1	325 (29.5)	202 (26.7)	528 (28.4)	
	N2	197 (17.9)	254 (33.6)	451 (24.2)	
	NX	179 (16.2)	177 (23.4)	357 (19.2)	
M stage	M0	737 (66.9)	215 (28.4)	953 (51.2)	< 0.001
	M1	173 (15.7)	428 (56.6)	602 (32.3)	
	MX	192 (17.4)	113 (14.9)	306 (16.4)	
Overall cancer stage	Stage 1	117 (10.6)	18 (2.4)	136 (7.3)	< 0.001
	Stage 2	199 (18.1)	58 (7.7)	257 (13.8)	
	Stage 3	323 (29.3)	104 (13.8)	427 (22.9)	
	Stage 4	173 (15.7)	428 (56.6)	602 (32.3)	
	Staging data incomplete	290 (26.3)	148 (19.6)	439 (23.6)	
Management strategy	Resection with anastomosis	566 (51.4)	95 (12.6)	662 (35.6)	< 0.001
	Resection with end stoma	310 (28.1)	120 (15.9)	430 (23.1)	
	Diverting stoma only	55 (5.0)	176 (23.3)	231 (12.4)	
	Laparotomy/laparoscopy without resection/stoma	18 (1.6)	25 (3.3)	43 (2.3)	
	No surgery	153 (13.9)	340 (45.0)	495 (26.6)	

*Note:* Values are *n* (%) unless stated otherwise. 3 patients had missing data on curative versus palliative intent excluded from the group‐wise comparisons.

The location of the primary tumor was most commonly in the left colon (837 [45.0%] of 1861), followed by the right colon (668 [35.9%]), and rectum (321 [17.2%]) with a small minority of patients with synchronous CRC (35 [1.9%], Figure 2).

Cancer staging data are presented in Table 1; 7.3% were Stage I (136 of 1861), 13.8% were Stage II (257 of 1861), 22.9% Stage III (427 of 1861), and 32.3% Stage IV (602 of 1861). The remaining 23.6% of patients had incomplete cancer staging with either missing nodal or metastatic status (439 of 1861). Of patients with incomplete staging, the majority had locally advanced T3 (172 [41.1%] of 439) or T4 disease (137 [32.8%] of 439).

### 3.1 Treatment Strategies

**FIGURE 3:**
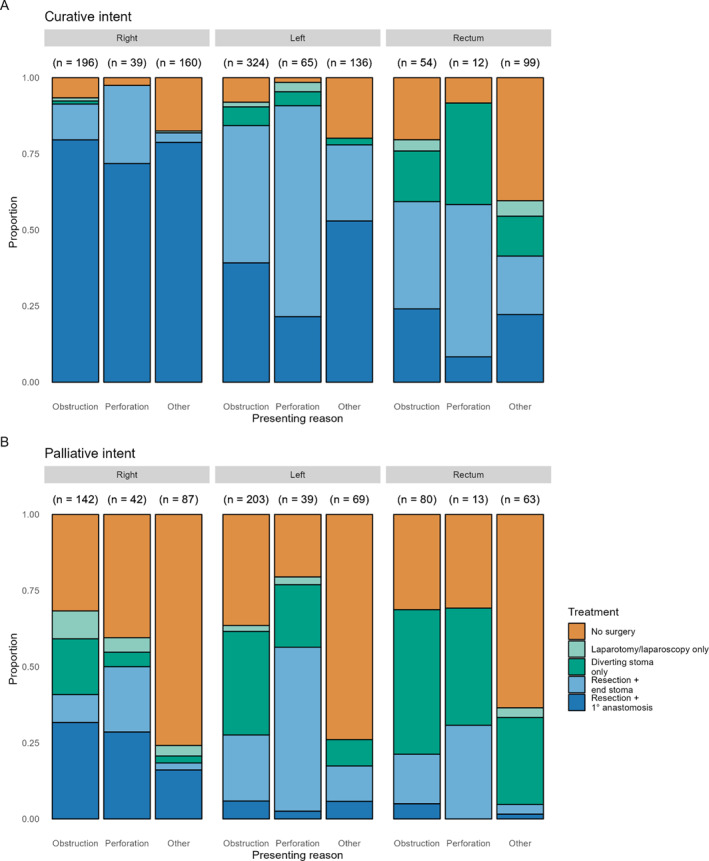
Inhospital management strategies of patients presenting acutely with colorectal cancer. (A) Patients managed with curative intent. (B) Patients with palliative intent. Patients with synchronous tumors are not displayed here.

In total, 59.2% of patients were managed with curative intent (1102 of 1861, Table 1). Patients managed with palliative intent were significantly older (71.0 vs. 69.0 years, *p* < 0.001), more comorbid with higher ASA grades (ASA III–V 52.2% vs. 40.3%, *p* < 0.001), higher clinical frailty scores (4.0 vs. 3.0, *p* < 0.001), and more likely underweight (BMI < 18.5 8.3% vs. 5.2%, *p* = 0.014). Stage 4 disease was more common in patients managed with palliative intent (56.6% vs. 15.7%, *p* < 0.001, Table 1). Management strategies stratified by treatment intent, location of CRC, and mode of presentation are summarized in Figure 3.

In right‐sided colon cancer (RCC) managed with curative intent, resection and primary anastomosis was the most commonly adopted surgical strategy (310 [78.5%] of 395, Supporting Information S1: Table 3). When patients with RCC were managed with palliative intent, 35% of patients (95 of 271) had palliative resection, whereas 11% (30 of 271, Supporting Information S1: Table 4) had a diverting stoma formed only. Colonic stenting was used infrequently in RCC regardless of the treatment of intent (5 [1.2%] of 395 curative RCC; 10 [3.7%] of 271 palliative RCC).

In left‐sided cancer (LCC) managed with curative intent, resection with end stoma (225 [42.9%] of 525, Supporting Information S1: Table 3) or resection with primary anastomosis was utilized at similar rates (213 [40.6%] of 525). The formation of a diverting stoma without resection only occurred in 5% of patients (26 of 525). When patients with LCC were managed palliatively, resection with end stoma (73 [23.5%] of 311) and diverting stoma formation only were utilized at similar rates (83 [26.7%] of 311, Supporting Information S1: Table 4). Colonic stenting was used in LCC more frequently in palliatively managed cases compared to curatively managed cases (38 [12.2%] of 311 palliative LCC, 24 [4.5%] of 525 curative LCC).

In rectal cancers managed with curative intent, management strategies were heterogeneously split between resection with primary anastomosis (36 [21.8%] of 165), resection with end stoma (44 [26.7%]), diverting stoma (26 [15.8%]), and nonoperative management (52, [31.5%], Supporting Information S1: Table 3). In rectal cancers managed with palliative intent, patients predominantly underwent diverting stoma formation only (61 [39.1%] of 156) or nonsurgical management (69 [44.2%] of 156, Supporting Information S1: Table 4).

### 3.2 Short‐Term Outcomes

**FIGURE 4:**
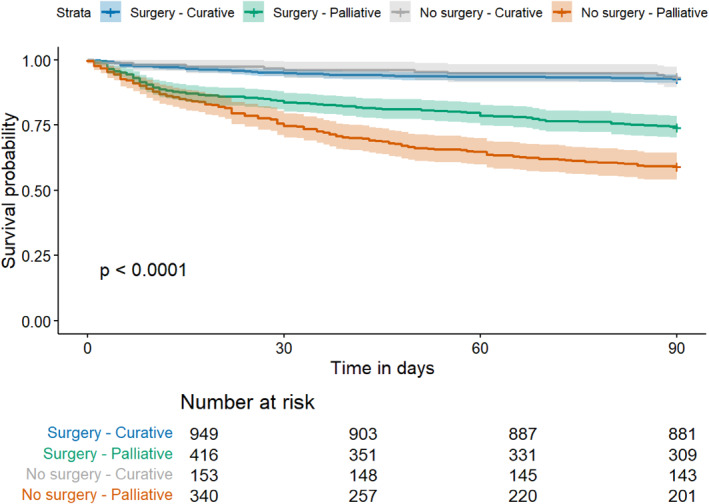
Survival curve stratified by treatment and treatment intent.

**FIGURE 5:**
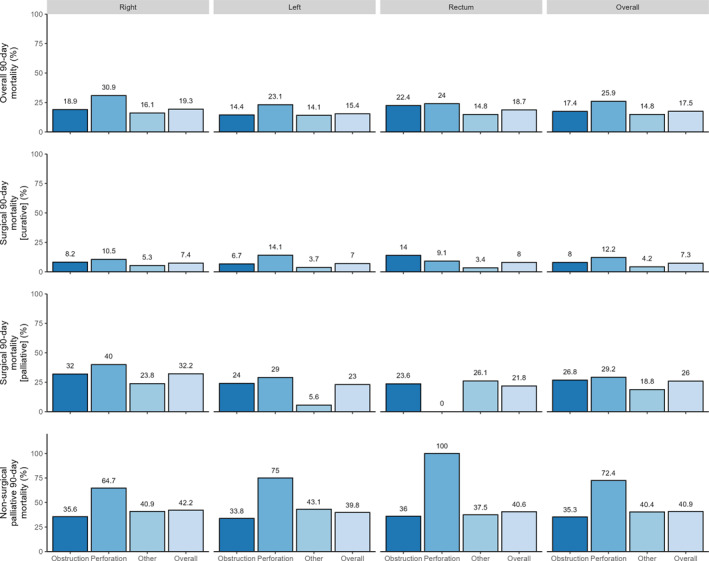
Overall unadjusted mortality stratified by location, reason for presentation, and management intent.

Kaplan–Meier curves stratified by the overall treatment strategy and intent is presented in Figure 4. Mortality stratified by patient presentation and by tumor location is displayed in Figure 5. Overall, 30‐ and 90‐day mortality in the entire cohort was 10.4% (194 of 1861) and 17.5% (326 of 1861), respectively. Mortality differed significantly between patients treated with curative surgical intent (69 [7.3%] of 949), palliative surgical intent (108 [26.0%] of 416), and nonoperative palliative intent (149 [40.9%] of 340).

### 3.3 Progression of Disease in Pre‐Existing CRC Patients

In this cohort, 42.8% of patients had a previous diagnosis of CRC and represented acutely (797 of 1861, Table 1). Of these patients, a significant portion was either awaiting elective surgery (273 [34.3%] of 797) or undergoing neoadjuvant chemotherapy/radiotherapy before surgery at the time of acute presentation (167 [21.0%] of 797). Another 27.6% of patients with previously diagnosed CRC presenting acutely were being palliatively managed without an operation [220 of 797].

In patients undergoing neoadjuvant chemotherapy/radiotherapy (*n* = 167), the most common reason for acute presentation was obstruction (80 [48.0%] of 167), gastrointestinal bleeding (41 [24.6%] of 167), and perforation (26 [15.6%] of 167).

A number of CRC patients with a previous diagnosis were subsequently transitioned to palliative treatment upon acute representation for progression of disease, including 16.8% of patients awaiting elective surgery (46 of 273) and 44.9% of (75 of 167) patients undergoing neoadjuvant chemotherapy or radiotherapy.

## 4 Discussion

This secondary analysis of the APOLLO prospective, multicenter study involving 1861 patients with acutely presenting CRC across 39 countries provided a snapshot of the global patterns of acute CRC presentation, management, and short‐term outcomes. Malignant bowel obstruction is the leading cause of acute hospital admission in patients with CRC. Strategies to manage acutely left‐sided colon cancer and rectal cancer are heterogeneous and varied. The overall 30‐ and 90‐day mortality in acutely presenting CRC was 10.4% and 17.5%, respectively; although patients with disease amenable to curative resection had a lower 90‐day mortality rate of 7.3%.

The overall short‐term mortality described in our cohort is comparable to other series ranging from 8% to 13% at 30 days [12, 13, 14]. Similarly, bowel obstruction remains the most common reason for emergency CRC presentations in this cohort like in other studies [14, 15], occurring in over 50% of patients admitted to hospital with acute CRC. Presentations secondary to bowel perforation were less common (11%) and with rates comparable to other series [14, 16]. Although previous studies have described the rate of obstruction in patients undergoing emergency CRC surgery as high as 70%–80% [16, 17], our study offers a more comprehensive overview of the rate of bowel obstruction by also including nonoperatively managed patients. Bowel obstruction and perforation are a hallmark of locally advanced CRC malignancy and have correspondingly poor outcomes [18]. The majority of patients in our cohort with obstruction or perforation presented with advanced CRC, often precluding curative surgical treatment.

Some patients with CRC presented while undergoing neoadjuvant therapy. Improved patient selection or monitoring could have plausibly prevented emergency admission while on neoadjuvant therapy. In locally advanced rectal adenocarcinoma, there is an increasing adoption of neoadjuvant chemoradiotherapy with neoadjuvant chemotherapy (total neoadjuvant chemoradiotherapy), with some patients achieving complete pathological responses [19]. However, nonresponders may experience worse oncological outcomes and delays in surgery [20]. Similarly in locally advanced colon cancer, although neoadjuvant chemotherapy in well‐selected patients is of oncological benefit [21, 22], a small portion of patients will develop bowel obstruction during their neoadjuvant course [23]. Patients who develop complications of neoadjuvant treatment, as demonstrated by the present cohort, can be subjected to emergency surgery, which is associated with significantly higher rates of complications and death in the postoperative period [24]. In an era where neoadjuvant treatment pathways are becoming increasingly complex, the risk of acute obstruction and complications needs to be a factor considered in multidisciplinary discussions when selecting patients for these treatments [23].

Surgical decision‐making in emergency CRC operations is clinically complex due to a multitude of factors including treatment intent, patient factors, and cancer characteristics. This is reflected in our data. Although the World Society of Emergency Surgery guidelines recommend that primary anastomosis be attempted in both obstructive right‐ and left‐sided cancers [5], our data suggest in practice this is highly variable. Despite data suggesting primary anastomosis in emergency left‐sided resection is safe in selected populations, associated with a higher stoma free survival and comparable mortality [25, 26, 27], our cohort demonstrated resection with end stoma remains the most common management strategy in obstructed or perforated left‐sided colon cancer. Existing studies have demonstrated the variation in this practice, with some published series stating that the majority of left‐sided obstructing cases are managed with resection and primary anastomosis [28], whereas others demonstrate equivalent utilization of resection with end stoma and resection with primary anastomosis [29]. Varied practice in primary anastomosis rate observed here and elsewhere may be in part explained by the varying availability of an on‐call colorectal surgeon. Subspecialty colorectal expertise may increase the chances that a patient receives a resection and primary anastomosis [29]. Furthermore, differential access to tertiary center axillary services such as interventional radiology, and postoperative intensive care support that represent a center's “capacity to rescue” may influence operative decisions to complete a primary anastomosis [30].

The World Society of Emergency Surgery guidelines recommend that in obstructive rectal cancer, patients undergo formation of a defunctioning stoma without resection in preparation for neoadjuvant treatment [5]. This practice is not widespread in our present cohort evidenced by the heterogeneous management of patients with acute rectal cancers. Varying protocols exist for neoadjuvant radiotherapy and chemotherapy given with the aim to downstage rectal cancers [19, 22, 31]. However, access to radiation oncology is varied worldwide [32], and limited access to radiotherapy may result in more patients receiving primary resection rather than a temporizing operation in preparation for neoadjuvant treatment.

This study presents one of the largest international prospective datasets on acutely presenting CRC patients. A major strength is its inclusion of both surgical and nonsurgical management, including palliative care and colonic stenting, across a diverse range of healthcare settings. However, there are limitations. The relative infrequency of emergency CRC presentations at individual hospitals resulted in a low average number of patients per site. Incomplete staging at the time of data collection limited complete analysis on the impact of cancer stage at diagnosis on outcomes, and staging data in nonoperatively managed cases were reliant on radiological staging only. The heterogeneity of the overall cohort makes overarching analysis challenging to interpret, and specific cohort questions will be explored separately. Finally, the study was unable to fully capture variations in healthcare systems, which inevitably influence decisions regarding emergency surgery versus expedited elective procedures.

In conclusion, this study characterizes the short‐term mortality rates of acutely managed CRC. Bowel obstruction remains the leading cause of acute presentation, associated with considerable mortality.

## Author Contributions

Please see Supporting Information S1 for full details of author contributions.

## Conflicts of Interest

The authors declare no conflicts of interest.

## Supporting information


Supporting Information S1

## Data Availability

The data that support the findings of this study are available on request from the corresponding author. The data are not publicly available due to privacy or ethical restrictions.
