# Supplementary material for: Acute Presentations of Colorectal Cancer: An International Prospective Snapshot on Management and Outcomes
Source: World J Surg. 2025 Oct 28;49(12):3368–77. doi: 10.1002/wjs.70106 (PMC12690004; doi:10.1002/wjs.70106)
Supplement: Supplementary file 1 — Supporting Information S1 [file WJS-49-3368-s001.docx]

# **Supplementary material**

**Supplementary table 1 - Contributions from each country**

| Country | Index colorectal cancer | Previously diagnosed colorectal cancer | Total cases |
| --- | --- | --- | --- |
| United Kingdom | 236 | 224 | 460 |
| Italy | 207 | 125 | 332 |
| Turkiye | 69 | 59 | 128 |
| New Zealand | 48 | 73 | 121 |
| Spain | 87 | 32 | 119 |
| Greece | 58 | 52 | 110 |
| Portugal | 45 | 29 | 74 |
| Australia | 49 | 19 | 68 |
| Bosnia and Herzegovina | 30 | 11 | 41 |
| Egypt | 18 | 23 | 41 |
| Libya | 19 | 17 | 36 |
| Malaysia | 24 | 4 | 28 |
| Russian Federation | 18 | 7 | 25 |
| Sri Lanka | 14 | 9 | 23 |
| Nigeria | 13 | 9 | 22 |
| Jordan | 14 | 4 | 18 |
| Germany | 13 | 4 | 17 |
| India | 6 | 10 | 16 |
| Morocco | 4 | 12 | 16 |
| Bulgaria | 4 | 11 | 15 |
| Switzerland | 11 | 3 | 14 |
| Sudan | 3 | 10 | 13 |
| Poland | 6 | 6 | 12 |
| Syria | 10 | 1 | 11 |
| Mexico | 7 | 3 | 10 |
| United States | 5 | 5 | 10 |
| Colombia | 3 | 6 | 9 |
| Iraq | 6 | 3 | 9 |
| Lithuania | 5 | 4 | 9 |
| Pakistan | 5 | 4 | 9 |
| Latvia | 5 | 3 | 8 |
| Qatar | 7 | 1 | 8 |
| South Africa | 2 | 6 | 8 |
| Algeria | 3 | 3 | 6 |
| Slovenia | 2 | 3 | 5 |
| Yemen | 4 | 1 | 5 |
| Croatia | 3 | 0 | 3 |
| Peru | 0 | 1 | 1 |
| Serbia | 1 | 0 | 1 |

##

**Supplementary table 2 - detailed reasons for presentation**

| **Variables** | | **Total** |
| --- | --- | --- |
| Total N (%) | | 1861 |
| Presenting reasons | Obstruction | 1015 (54.5) |
|  | Perforation | 212 (11.4) |
|  | GI bleed | 386 (20.7) |
|  | Other abdominal pain | 114 (6.1) |
|  | Bowel habit change | 42 (2.3) |
|  | Constitutional symptoms (weight loss, fatigue, lack of appetite) | 18 (1.0) |
|  | Sepsis | 9 (0.5) |
|  | Abscess/fistula | 15 (0.8) |
|  | Other | 50 (2.7) |

##

**Supplementary table 3 - Patients with curatively managed cancers by location**

| **Variable** | | **Right** | **Left** | **Rectum** | **Synchronous** | **Total** |
| --- | --- | --- | --- | --- | --- | --- |
| Total N (%) | | 395 (35.8) | 525 (47.6) | 165 (15.0) | 17 (1.5) | 1102 |
| Age | Median (IQR) | 71.0 (59.0 to 80.0) | 68.0 (57.0 to 77.0) | 65.0 (57.0 to 74.0) | 65.0 (55.0 to 76.0) | 69.0 (58.0 to 77.0) |
| Sex | Female | 193 (48.9) | 236 (45.0) | 49 (29.7) | 7 (41.2) | 485 (44.0) |
|  | Male | 202 (51.1) | 289 (55.0) | 116 (70.3) | 10 (58.8) | 617 (56.0) |
| BMI | < 18.5 | 23 (5.8) | 22 (4.2) | 12 (7.3) | 1 (5.9) | 58 (5.3) |
|  | 18.5 - 24.9 | 161 (40.8) | 222 (42.3) | 76 (46.1) | 5 (29.4) | 464 (42.1) |
|  | 25 - 29.9 | 136 (34.4) | 143 (27.2) | 43 (26.1) | 6 (35.3) | 328 (29.8) |
|  | 30 - 39.9 | 43 (10.9) | 79 (15.0) | 19 (11.5) | 3 (17.6) | 144 (13.1) |
|  | >40 | 7 (1.8) | 11 (2.1) | 2 (1.2) | 1 (5.9) | 21 (1.9) |
|  | (Missing) | 25 (6.3) | 48 (9.1) | 13 (7.9) | 1 (5.9) | 87 (7.9) |
| ASA | I-II | 208 (52.7) | 304 (57.9) | 99 (60.0) | 7 (41.2) | 618 (56.1) |
|  | III-V | 172 (43.5) | 205 (39.0) | 57 (34.5) | 10 (58.8) | 444 (40.3) |
|  | Not recorded | 15 (3.8) | 16 (3.0) | 9 (5.5) | 0 (0.0) | 40 (3.6) |
| Clinical Frailty Score (1-9) | Median (IQR) | 3.0 (2.0 to 4.0) | 3.0 (2.0 to 4.0) | 3.0 (2.0 to 4.0) | 3.0 (2.0 to 4.0) | 3.0 (2.0 to 4.0) |
| Smoking Status | Current | 52 (13.2) | 82 (15.6) | 30 (18.2) | 4 (23.5) | 168 (15.2) |
|  | Ex-smoker | 89 (22.5) | 96 (18.3) | 33 (20.0) | 4 (23.5) | 222 (20.1) |
|  | Never smoked | 175 (44.3) | 251 (47.8) | 68 (41.2) | 5 (29.4) | 499 (45.3) |
|  | Unknown | 79 (20.0) | 96 (18.3) | 34 (20.6) | 4 (23.5) | 213 (19.3) |
| Previous abdominal surgery | No | 242 (61.3) | 360 (68.6) | 90 (54.5) | 8 (47.1) | 700 (63.5) |
|  | Yes | 152 (38.5) | 165 (31.4) | 75 (45.5) | 9 (52.9) | 401 (36.4) |
|  | (Missing) | 1 (0.3) | 0 (0.0) | 0 (0.0) | 0 (0.0) | 1 (0.1) |
| Cancer status | First presentation of cancer | 271 (68.6) | 354 (67.4) | 63 (38.2) | 9 (52.9) | 697 (63.2) |
|  | Known cancer | 124 (31.4) | 171 (32.6) | 102 (61.8) | 8 (47.1) | 405 (36.8) |
| Presenting reason | Obstruction | 196 (49.6) | 324 (61.7) | 54 (32.7) | 4 (23.5) | 578 (52.5) |
|  | Perforation | 39 (9.9) | 65 (12.4) | 12 (7.3) | 2 (11.8) | 118 (10.7) |
|  | Other | 160 (40.5) | 136 (25.9) | 99 (60.0) | 11 (64.7) | 406 (36.8) |
| T stage | T1 | 47 (11.9) | 37 (7.0) | 17 (10.3) | 0 (0.0) | 101 (9.2) |
|  | T2 | 56 (14.2) | 63 (12.0) | 37 (22.4) | 4 (23.5) | 160 (14.5) |
|  | T3 | 157 (39.7) | 265 (50.5) | 59 (35.8) | 9 (52.9) | 490 (44.5) |
|  | T4 | 132 (33.4) | 157 (29.9) | 51 (30.9) | 4 (23.5) | 344 (31.2) |
|  | (Missing) | 3 (0.8) | 3 (0.6) | 1 (0.6) | 0 (0.0) | 7 (0.6) |
| N stage | N0 | 143 (36.2) | 205 (39.0) | 45 (27.3) | 8 (47.1) | 401 (36.4) |
|  | N1 | 110 (27.8) | 156 (29.7) | 53 (32.1) | 6 (35.3) | 325 (29.5) |
|  | N2 | 74 (18.7) | 75 (14.3) | 47 (28.5) | 1 (5.9) | 197 (17.9) |
|  | NX | 68 (17.2) | 89 (17.0) | 20 (12.1) | 2 (11.8) | 179 (16.2) |
| M stage | M0 | 269 (68.1) | 347 (66.1) | 110 (66.7) | 11 (64.7) | 737 (66.9) |
|  | M1 | 52 (13.2) | 82 (15.6) | 36 (21.8) | 3 (17.6) | 173 (15.7) |
|  | MX | 74 (18.7) | 96 (18.3) | 19 (11.5) | 3 (17.6) | 192 (17.4) |
| Overall cancer stage | Stage 1 | 44 (11.1) | 46 (8.8) | 24 (14.5) | 3 (17.6) | 117 (10.6) |
|  | Stage 2 | 68 (17.2) | 111 (21.1) | 17 (10.3) | 3 (17.6) | 199 (18.1) |
|  | Stage 3 | 120 (30.4) | 141 (26.9) | 58 (35.2) | 4 (23.5) | 323 (29.3) |
|  | Stage 4 | 52 (13.2) | 82 (15.6) | 36 (21.8) | 3 (17.6) | 173 (15.7) |
|  | Staging data incomplete | 111 (28.1) | 145 (27.6) | 30 (18.2) | 4 (23.5) | 290 (26.3) |
| Management strategy | Resection with anastomosis | 310 (78.5) | 213 (40.6) | 36 (21.8) | 7 (41.2) | 566 (51.4) |
|  | Resection with end stoma | 38 (9.6) | 225 (42.9) | 44 (26.7) | 3 (17.6) | 310 (28.1) |
|  | Diverting stoma only | 2 (0.5) | 26 (5.0) | 26 (15.8) | 1 (5.9) | 55 (5.0) |
|  | Laparotomy/  laparoscopy without resection/  stoma | 3 (0.8) | 7 (1.3) | 7 (4.2) | 1 (5.9) | 18 (1.6) |
|  | No surgery | 42 (10.6) | 54 (10.3) | 52 (31.5) | 5 (29.4) | 153 (13.9) |

##

**Supplementary table 4 - Patients with palliative managed cancers by location**

| **Variable** | | **Right** | **Left** | **Rectum** | **Synchronous** | **Total** |
| --- | --- | --- | --- | --- | --- | --- |
| Total N (%) | | 271 (35.8) | 311 (41.1) | 156 (20.6) | 18 (2.4) | 756 |
| Age | Median (IQR) | 75.0 (64.0 to 84.0) | 69.0 (58.5 to 79.5) | 69.0 (58.0 to 78.0) | 76.0 (65.2 to 82.0) | 71.0 (60.0 to 82.0) |
| Sex | Female | 137 (50.6) | 137 (44.1) | 65 (41.7) | 7 (38.9) | 346 (45.8) |
|  | Male | 134 (49.4) | 174 (55.9) | 91 (58.3) | 11 (61.1) | 410 (54.2) |
| BMI | < 18.5 | 23 (8.5) | 21 (6.8) | 18 (11.5) | 1 (5.6) | 63 (8.3) |
|  | 18.5 - 24.9 | 91 (33.6) | 127 (40.8) | 72 (46.2) | 6 (33.3) | 296 (39.2) |
|  | 25 - 29.9 | 67 (24.7) | 68 (21.9) | 41 (26.3) | 5 (27.8) | 181 (23.9) |
|  | 30 - 39.9 | 50 (18.5) | 47 (15.1) | 8 (5.1) | 1 (5.6) | 106 (14.0) |
|  | >40 | 3 (1.1) | 7 (2.3) | 5 (3.2) | 1 (5.6) | 16 (2.1) |
|  | (Missing) | 37 (13.7) | 41 (13.2) | 12 (7.7) | 4 (22.2) | 94 (12.4) |
| ASA | I-II | 94 (34.7) | 129 (41.5) | 71 (45.5) | 4 (22.2) | 298 (39.4) |
|  | III-V | 149 (55.0) | 158 (50.8) | 74 (47.4) | 14 (77.8) | 395 (52.2) |
|  | Not recorded | 28 (10.3) | 24 (7.7) | 11 (7.1) | 0 (0.0) | 63 (8.3) |
| Clinical Frailty Score (1-9) | Median (IQR) | 5.0 (3.0 to 6.0) | 4.0 (3.0 to 6.0) | 4.0 (3.0 to 6.0) | 6.0 (4.0 to 7.0) | 4.0 (3.0 to 6.0) |
| Smoking Status | Current | 35 (12.9) | 41 (13.2) | 21 (13.5) | 2 (11.1) | 99 (13.1) |
|  | Ex-smoker | 58 (21.4) | 68 (21.9) | 28 (17.9) | 6 (33.3) | 160 (21.2) |
|  | Never smoked | 114 (42.1) | 132 (42.4) | 59 (37.8) | 6 (33.3) | 311 (41.1) |
|  | Unknown | 64 (23.6) | 70 (22.5) | 48 (30.8) | 4 (22.2) | 186 (24.6) |
| Previous abdominal surgery | No | 160 (59.0) | 190 (61.1) | 83 (53.2) | 12 (66.7) | 445 (58.9) |
|  | Yes | 111 (41.0) | 121 (38.9) | 73 (46.8) | 6 (33.3) | 311 (41.1) |
| Cancer status | First presentation of cancer | 148 (54.6) | 160 (51.4) | 48 (30.8) | 8 (44.4) | 364 (48.1) |
|  | Known cancer | 123 (45.4) | 151 (48.6) | 108 (69.2) | 10 (55.6) | 392 (51.9) |
| Presenting reason | Obstruction | 142 (52.4) | 203 (65.3) | 80 (51.3) | 12 (66.7) | 437 (57.8) |
|  | Perforation | 42 (15.5) | 39 (12.5) | 13 (8.3) | 0 (0.0) | 94 (12.4) |
|  | Other | 87 (32.1) | 69 (22.2) | 63 (40.4) | 6 (33.3) | 225 (29.8) |
| T stage | T1 | 19 (7.0) | 10 (3.2) | 2 (1.3) | 0 (0.0) | 31 (4.1) |
|  | T2 | 21 (7.7) | 22 (7.1) | 9 (5.8) | 2 (11.1) | 54 (7.1) |
|  | T3 | 83 (30.6) | 102 (32.8) | 55 (35.3) | 4 (22.2) | 244 (32.3) |
|  | T4 | 139 (51.3) | 172 (55.3) | 87 (55.8) | 12 (66.7) | 410 (54.2) |
|  | (Missing) | 9 (3.3) | 5 (1.6) | 3 (1.9) | 0 (0.0) | 17 (2.2) |
| N stage | N0 | 41 (15.1) | 53 (17.0) | 25 (16.0) | 4 (22.2) | 123 (16.3) |
|  | N1 | 70 (25.8) | 84 (27.0) | 46 (29.5) | 2 (11.1) | 202 (26.7) |
|  | N2 | 88 (32.5) | 104 (33.4) | 55 (35.3) | 7 (38.9) | 254 (33.6) |
|  | NX | 72 (26.6) | 70 (22.5) | 30 (19.2) | 5 (27.8) | 177 (23.4) |
| M stage | M0 | 65 (24.0) | 84 (27.0) | 64 (41.0) | 2 (11.1) | 215 (28.4) |
|  | M1 | 161 (59.4) | 184 (59.2) | 72 (46.2) | 11 (61.1) | 428 (56.6) |
|  | MX | 45 (16.6) | 43 (13.8) | 20 (12.8) | 5 (27.8) | 113 (14.9) |
| Overall cancer stage | Stage 1 | 10 (3.7) | 5 (1.6) | 2 (1.3) | 1 (5.6) | 18 (2.4) |
|  | Stage 2 | 12 (4.4) | 32 (10.3) | 14 (9.0) |  | 58 (7.7) |
|  | Stage 3 | 27 (10.0) | 40 (12.9) | 36 (23.1) | 1 (5.6) | 104 (13.8) |
|  | Stage 4 | 161 (59.4) | 184 (59.2) | 72 (46.2) | 11 (61.1) | 428 (56.6) |
|  | Staging data incomplete | 61 (22.5) | 50 (16.1) | 32 (20.5) | 5 (27.8) | 148 (19.6) |
| Management strategy | Resection with anastomosis | 71 (26.2) | 17 (5.5) | 5 (3.2) | 2 (11.1) | 95 (12.6) |
|  | Resection with end stoma | 24 (8.9) | 73 (23.5) | 19 (12.2) | 4 (22.2) | 120 (15.9) |
|  | Diverting stoma only | 30 (11.1) | 83 (26.7) | 61 (39.1) | 2 (11.1) | 176 (23.3) |
|  | Laparotomy/  laparoscopy without resection/  stoma | 18 (6.6) | 5 (1.6) | 2 (1.3) | 0 (0.0) | 25 (3.3) |
|  | No surgery | 128 (47.2) | 133 (42.8) | 69 (44.2) | 10 (55.6) | 340 (45.0) |

**Appendix 1: Authorship list**

**WRITING GROUP**

William Xu**, Chris Varghese, Daoud Chaudhry, Mustafa Ege Seker, Noor Essa, Ian Bissett, Susan Moug, Wal Baraza, Sue Blackwell, Dion Morton, Francesco Pata, Gianluca Pellino, Dimitri Nepogodiev, Umar Saeed, James Glasbey, Alessandro Sgrò^+^, Muhammad Elhadi^+^

**First author

^+^Joint senior author

**CRediT statement**

Conceptualisation: WX, CV, DC, MES, NE, MSA, MS, SO, AT, IS, LK, ME

Methodology: WX, CV, DC, MES, NE, MSA, MS, SO, AT, IS, LK, ME

Software: WX, CV, DC, JG

Validation: WX, CV, DC, JG

Formal analysis: WX, CV, DC

Investigation: WX, CV, DC, MES, NE, IB, SM, WB, SB, DM

Resources: WX, CV, DC, MES, NE, IB, SM, WB, SB, DM

Data Curation: All collaborators

Writing - Original Draft: WX, CV, DC, MES, NE, IB, SM, WB, SB, DM, FP, GP, DN, US, JG, AS, ME

Writing - Review and Editing: WX, CV, DC, MES, NE, IB, SM, WB, SB, DM, FP, GP, DN, US, JG, AS, ME

Visualisation: WX,CV, DC

Supervision: IB, SM, WB, SB, DM, FP, GP, DN, US, JG, AS, ME

Project administration: WX, CV, DC, MES, NE, MSA, MS, SO, AT, IS, LK, ME

Funding acquisition: No funding for this study

**STUDY MANAGEMENT GROUP**

William Xu, Chris Varghese, Daoud Chaudhry, Mustafa Ege Seker, Noor Essa, Mafalda Sampaio Alves, Moritz Steinruecke, Setthasorn Ooi, Adam Turňa, Irene Santos, Laura Kehoe, Muhammad Elhadi

**EXPERT ADVISORY GROUP**

Ian Bissett, Stephen Chapman, Ruth Blanco Colino, James Glasbey, Susan Moug, Dion Morton, Wal Baraza, Sue Blackwell, Dimitri Nepogodiev, Francesco Pata, Gianluca Pellino, Peter Pockney, Alessandro Sgrò,

**DATA ANALYSIS**

William Xu, Chris Varghese

**NATIONAL LEADS**

Amanda Dawson, Loranne Gaborit, Sarah Goh (Australia), Samir Delibegovic (Bosnia and Herzegovina), Arwa Mohamad (Bulgaria), Jakov Mihanovic (Croatia), Daniela Arbeláez-Lelion (Colombia), Daniela Merz (Germany), OrestisIoannidis, Argyrios Ioannidis (Greece), Sahana Bopparaju (Telangana-India), Francesco Pata, Gianluca Pellino (Italy), Hoshika Tharni Sivapalan (Latvia), Muhammed Elhadi (Libya and MENA), Albertas Dauksa (Lithuania), Andee Dzulkarnaen Zakaria (Malaysia), Luis Adrian Alvarez-Lozada (Mexico), Anthony Lin, Shuba Kosna (New Zealand), Ademola Adeyeye (Nigeria), Umar Saeed (Pakistan), Irène Santos, José Guilherme Gonçalves-Nobre, Mafalda Sampaio-Alves (Portugal), Anastasia Novikova (Russian Federation), Melik Kağan Aktaş, Mustafa Deniz Tepe (Türkiye), Jurij Ales Kosir (Slovenia), Neoleen Van Staden (South Africa),Ruth Blanco-Colino (Spain),Cristiana Riboni, Dimitri Christoforidis (Switzerland), Moritz Steinruecke, Setthasorn Ooi, Louise Ko (United Kingdom)

**REGIONAL LEADS (UK/IRELAND/ITALY ONLY)**

Kirsty Luo-Yng Tay (Aberdeen); Fatimah Khan (Anglia Ruskin University); Ronald Hang Kin Nam (Aston); Aleksandra Lopuszko (Barts and the London (QMUL)); Patrick Keane (Belfast (QUB)); Warda Jamshaid (Birmingham ); Pierre Jean-Marie (Brighton & Sussex); Runqing Su (Bristol); Vian Omar (Buckingham); Ellen Fung (Cambridge); Maryam Jamshaid (Cardiff); Ashna Ashpak (Central Lancashire); Xianghan Zheng (Cork); Ned Quirke (Dublin (UCD)); Tasnim Kouli (Dundee); Yuk Wing Liza Chong (Edinburgh); Alexandra Sebastiao (Exeter); Nahl Iftikhar (Glasgow); Mitchel Shula (Hull York ); Kavyesh Vivek (Imperial); Balamrit Singh Sokhal (Keele); Anthony Siu (Kings); Hamzah Ahmin (Lancaster); Ankit Gupta (Leeds); Sanjana Shaunak (Leicester); Arooj Qaiser (Lincoln (Nottingham)); Shubhi Gupta (Liverpool); Ayesha Qureshi (Manchester); Richard Huynh (Newcastle); Rhea Suribhatla (Oxford); Sam Ghaznavi (Peninsula (Plymouth)); Sanjana Mehrotra (Sheffield); Kiran Stowell (Southampton); Kofi Cox (St George's); Hermes Manos (St. Andrews); Roshni Patel (Swansea); K.Ewomazino Oderoha (Trinity College Dublin); Raian Jaibaji (UCL); Ellisa Baggott (UEA (Norwich)); Semhar Abraha (Warwick), Cristiana Riboni, Salomone di Saverio, Alice Gori, Vinicio Mosca, Mauro Podda, Matteo Rottoli, Alessandro Sgrò (Italy)

**HOSPITAL LEADS**

**Algeria**: Anisse Tidjane (Ehu-1st November 1954).

**Australia**: David Proud (Austin Hospital); Shane Zhang (Calvary Mater Newcastle); Gemma Qian, Amanda Dawson(Gosford Hospital); Edward Zhang (John Hunter Hospital); Antonio Barbaro (Lyell McEwin Hospital); Madison Lowe (Mount Gambier And Districts Health Service); Madhavi-Priya Singh (Northern Hospital); Luke Traeger, Tarik Sammour (Royal Adelaide Hospital); Talia Shepherd (Royal Perth Hospital); Mary Theophilus (St John Of God Midland Public And Private Hospital); Qiwen Wang (The Queen Elizabeth Hospital); Richard G McGee (Wyong Public Hospital).

**Bosnia And Herzegovina**: Merima Kruščica, Mirhan Salibašić (Clinical Center University Of Sarajevo); Haris Kuralić (University Clinical Center Tuzla).

**Bulgaria**: Dimitar Hadzhiev (Umhat Sveti Georgi); Martin Karamanliev (University Hospital Dr Georgi Stranski).

**Colombia**: Daniela Arbelaez Lelion (Hospital Pablo Tobón Uribe).

**Croatia**: Jakov Mihanovic (Zadar General Hospital).

**Egypt**: Ahmed Sabry (Alexandria Main University Hospital); Ahmed M. Abbas (Assiut University Hospital); Sarah Abdelmohsen (Aswan University Hospital); Ahmed Saber Mohamed Abdelrahman (Giza International Hospital); Khaled Abdelwahab (Oncology Center Mansoura University); Mohamed Elbahnasawy (Tanta University Hospital); Bassam Fahmy (The Memorial Soaad Kafafi University Hospital).

**Germany**: Daniel Reim (Technical University of Munich, School of Medicine and Health, TUM University Hospital, Department of Surgery); Gregor Massoth (University Hospital Bonn); Ulrich Ronellenfitsch (University Hospital Halle).

**Greece**: Dimitrios Korkolis (Agios Savvas Anticancer Hospital); Christos Chouliaras (Athens Medical Center); Dimitrios Manatakis (Athens Naval And Veterans Hospital); Theodoros A Sidiropoulos (Attikon University General Hospital); Maria Sotiropoulou (Evaggelismos General Hospital); Prokopis Christodoulou (General Hospital Asklepieio Voulas); Francesk Mulita (General University Hospital Of Patras); Orestis Ioannidis (George Papanikolaou General Hospital Of Thessaloniki); Maximos Frountzas (Hippocratio General Hospital); Nikolaos Machairas (Laiko University Hospital); Ioannis Katsaros (Metaxa Cancer Hospital); Michael Spartalis (Sotiria General Hospital Of Thoracic Diseases); Konstantinos Lasithiotakis (University Hospital Of Heraklion Crete).

**India**: Yashwant Sakaray (Post Graduate Institute of Medical Education and Research).

**Iraq**: Ali Kadhim (Al-hussien Medical City)

**Ireland:** Ireland: Ned Quirke (St Vincent's University Hospital); Kevin Ewomazino Oderoha (St James's Hospital)

**Italy**: Stefano Piero Bernardo Cioffi (Asst Grande Ospedale Metropolitano Niguarda); Bruno Nardo (Azienda Ospedaliera Di Cosenza); Nicola Passuello (Azienda Ospedaliera Di Padova); Giulia Turri (Azienda Ospedaliera Universitaria Integrata Di Verona); Massimiliano Veroux (Azienda Ospedaliero- Universitaria Policlinico San Marco); Mauro Podda (Cagliari University Hospital); Alan Biloslavo (Cattinara University Hospital); Alessandra Marano (A.O.U. Città della Salute e della Scienza di Torino); Marco Amisano (Irccs Ospedale Policlinico San Martino); Daniela Rega (Istituto Nazionale Tumori Fondazione); Pasquale Cianci (Lorenzo Bonomo); Luca Cardinali (Madonna Del Soccorso Hospital); Daunia Verdi (Mirano Hospital); Fabrizio D'acapito (Morgagni-Pierantoni Hospital AUSL Romagna); Alessandro Broglia (Ospedale Civile Di Voghera); Francesco Maria Carrano (Ospedale Di Circolo Di Busto Arsizio); Giovanni Tarchi (Ospedale Di Legnano); Marco De Prizio (Ospedale San Donato Usl Toscana Sud Est); Nicolò Tamini (IRCCS San Gerardo dei Tintori - Monza); Andrea-Pierre Luzzi (Ospedale Villa Scassi); Vincenzo Lizzi (Ospedali Riuniti Azienda Ospedaliera Universitaria Foggia); Pierfrancesco Lapolla (Policlinico Umberto I); Francesco Fleres (University of Messina, Messina); Gaetano Poillucci (San Matteo Degli Infermi); Marco Clementi (San Salvatore Hospital, University of L’Aquila); Valeria Tonini (Santa Annunziata Hospital); Giacomo Calini (Ospedale Santa Maria della Misericordia di Udine); Nicola Cillara (Santissima Trinità - Ats Sardegna); Matteo Desio (University Of Insubria)

**Jordan**: Majedah Hmeidan (Al-Tafilah New Hospital); Bourhan Alrayes (Islamic Hospital); Almu'atasim Khamees (Jordan University Hospital); Mahmoud Mahafdah (King Abdullah University Hospital/ Jordan University Of Science And Technology); Samah Alananzeh (Princess Basma Hospital).

**Latvia**: Nityanand Jain (Pauls Stradins Clinical University Hospital)

**Libya**: Arwa Kara (Benghazi Medical Center); Hamida El Magrahi (Crown Health Care Clinical Team); Hibah Bileid Bakeer (Gharyan Central Hospital); Ayyah Emran (Tobruk Medical Center); Eman Abdulwahed (Tripoli Central Hospital); Mohamed Alsori (Tripoli Medical Center/ Tripoli University Hospital); Najat Ben Hasan (Zliten Teaching Hospital).

**Lithuania**: Kristina Marcinkevičiūtė (Vilnius University Hospital Santaros Klinikos).

**Malaysia**: Andee Dzulkarnaen Zakaria (School of Medical Sciences & Universiti Sains Malaysia Specialist Hospital); April Camilla Roslani (University Malaya Medical Centre).

**Mexico**: Francisco Emmanuel Alvarez Bautista (Hospital General Dr. Manuel Gea González); Danilo Tueme De La Peña (Instituto Nacional De Ciencias Médicas Y Nutrición 'Salvador Zubirán').

**Morocco**: Samia Errami (Hopital Ibn Tofail); Samia Kessab (Institut National D'oncologie).

**New Zealand**: Ashley Pereira, Wal Baraza (Auckland City Hospital); William Ju, Tamara Glyn (Christchurch Hospital); Avinash Sathiyaseelan, Sze Lin Peng (Middlemore Hospital); Cheuk Lam Jeffrey Lui, Xiao Shen Hu, Siraj Rajaratnam (North Shore Hospital); Edmund Leung (Taranaki Base Hospital); Binura Lekamalage, Jeremy Rossaak (Tauranga Hospital); Niki Kau, Jesse Fischer (Waikato Hospital); Sarah Rennie (Wairarapa Hospital); Mairarangi Haimona, Anthony Lin (Wellington Regional Hospital); Matthew McGuinness, Christopher Harmston (Whangarei Hospital).

**Nigeria**: Abubakar Bala Muhammad (Aminu Kano Teaching Hospital); Nurudeen Akinbami (University College Hospital); Matthew Bojuwoye (University Of Ilorin Teaching Hospital).

**Pakistan**: Warda Ahmed (Aga Khan University).

**Poland**: Jan Nicikowski (University Hospital of Karol Marcinkowski in Zielona Góra).

**Portugal**: Sofia Reis (Centro Hospitalar Barreiro Montijo); Daniela Martins (Centro Hospitalar De Trás-Os-Montes E Alto Douro); Marta Costa (Centro Hospitalar E Universitário De Coimbra - Hospital Geral); Penélope Correia (Centro Hospitalar Entre O Douro E Vouga); Rita Galama (Centro Hospitalar Médio Tejo); Beatriz Mendes (Centro Hospitalar Universitario Do Algarve - Unidade De Portimão); Ana Rita Loureiro (Hospital Das Caldas Da Rainha - Centro Hospitalar Do Oeste); Sonia Fortuna Martins (Hospital De Santarem); Joana Bolota (Hospital Do Espirito Santo); Alberto Silva (Hospital Do Litoral Alentejano); Madalena Trindade (Hospital Garcia De Orta); Lígia Freire (Unidade Local De Saude De Matosinhos - Hospital Pedro Hispano).

**Qatar**: Leena Aboidris (Hamad General Hospital).

**Russian Federation**: Sergey Efetov (Im Sechenov First Moscow State Medical University); Aleksandr Butyrskii (Municipal Emegency Hospital No.6); Alexey Yanishev (Privolzhsky Research Medical University).

**South Africa**: Margot Flint (Groote Schuur Hospital).

**Spain**: Zutoia Balciscueta (Hospital Arnau De Vilanova); David Moro-Valdezate (Hospital Clínico Universitario De Valencia); Ana Maria Minaya Bravo (Hospital Del Henares); Silvia Pérez-Ajates (Hospital General Universitario Gregorio Marañón); Fernando Mendoza-Moreno (Hospital Universitario Principe De Asturias); Noelia Ibáñez (Hospital Universitario Virgen De La Arrixaca); Felipe Pareja Ciuró (Hospital Universitario Virgen Del Rocio); Jorge Sancho-Muriel (Hospital Universitario Y Politécnico La Fe); Aitor Landaluce-Olavarria (Hospital Urduliz); Ana María Camacho Oliva (Puerta del Mar University Hospital); Mercedes Estaire Gómez (Severo Ochoa University Hospital).

**Sri Lanka**: Umesh Jayarajah (Colombo South Teaching Hospital); Sanjeewa Seneviratne (National Hospital Of Sri Lanka).

**Sudan**: Mohamed Musa Yassin (Gadarif Teaching Hospital); Essam Eldien Abuobaida (Ribat University Hospital).

**Switzerland**: Vaihere Delaune (Geneva University Hospitals); Joanna Naemi Marx (Kantonsspital Olten); Jörn-Markus Gass (Luzerner Kantonsspital)

**Syrian Arab Republic**: Mohamad Klib (Al-Mouwasat University Hospital); Anwar Chammout (Aleppo University Hospital).

**Turkey**: Gokalp Kagan Kurtoglu (Acibadem Altunizade Hospital); Emre Tuzuner (Acibadem Maslak Hospital); Beste Yıldırım (Dokuz Eylul Univ. Hospital); Kaan Okumuş (Ege University Hospital); Banu Yigit (Elazig Fethi Sekin City Hospital); Abdullah Emre Askin (Istanbul Medipol University Hospital); Ergin Erginöz (Istanbul Universty - Cerrahpaşa Medical Faculty); Ozgem Uysal (Izmir Katip Celebi University Faculty of Medicine); Aras Emre Canda (Acibadem Izmir Kent Hospital); Ayse Nilufer Yuzgec (Karadeniz Technical University Farabi Hospital); İbrahim Halil Özata (Koç University Medical School); Muhammed Enes Tasci (Marmara University School of Medicine); Nurhilal Kızıltoprak (Sultan 2. Abdülhamid Han Research and Training Hospital); Merve Yaren Kayabaş (Trakya University Hospital); Kemal Erdinç Kamer (University Of Health Sciences Tepecik Training And Research Hospital); Bulent Citgez (Uskudar University Faculty Of Medicine).

**United Kingdom:** Kirsty Luo-Yng Tay (Aberdeen Royal Infirmary); Alexia Farrugia (Sandwell General Hospital), Jessica Chang (Good Hope Hospital), Sharad Karandikar (Heartlands Hospital), Smaragda Gkolia (Royal London Hospital), Tara Chan-a-sue (Belfast City Hospital), Aine McGettigan (Royal Victoria Hospital), Ellen Dunlop (Ulster Hospital Dundonald), Grace Doherty (Antrim Area Hospital- Northern Health And Social Care Trust), Feargal McKey (Craigavon Area Hospital), Shabnam Cyclewala (Bristol Royal Infirmary), Vimaladhithan Mahendran (Yeovil District Hospital), Isabella Sawyer (Royal United Hospital Bath), Thomas Hibbs (Gloucestershire Royal Hospital), Hayden Simmons (Western General Hospital), Felicity Greenfield (Great Western Hospital), Pierre Jean-Marie (Royal Sussex County Hospital), Frances Dixon (Milton Keynes University Hospital), James Everson (Stoke Mandeville), Zafar Shahbaz (South Warwickshire NHS Foundation Trust), Fathima Manaal (Addenbrooke's Hospital), Kwan Wai Fung (Peterborough City Hospital), Jianing You (Hinchingbrooke Hospital), Cheuk Man Lam (West Suffolk Hospital), Emily Smith (Bedford Hospital), Ashna Ashpak (Royal Blackburn Hospital), Jenna Cook (Ninewells Hospital), Liza YW Chong (Western General Hospital), Melissa Bennett (Royal Devon And Exeter Hospital), Aris Alexiadis (Royal Cornwall Hospital), Hwei Jene Ng (Royal Alexandra Hospital), Mishal Shahid (Inverclyde Royal Hospital), Kavyesh Vivek (West Middlesex University Hospital), Kavyesh Vivek (Chelsea And Westminster Hospital), Kavyesh Vivek (St Mary's Hospital), Kavyesh Vivek (Charing Cross Hospital), Sadhasivam Ramasamy (University Hospitals Of North Midlands), Kareem Omran (Guy's And St Thomas' Hospitals), Ruairi Doherty (Furness General Hospital), Aqib Khan (Royal Lancaster Infirmary), Arooj Qaiser (Lincoln County Hospital), Jahnavi Kalvala (Queens Medical Centre), Michaela Silver (St James's University Hospital Leeds), Matthew Fok (Countess Of Chester Hospital), Rachael Clifford (Aintree University Hospital), Shubhi Gupta (Wirral University Teaching Hospital), Ammarah Ughratdar (Royal Preston Hospital), Oluwatobi Adegboye (Salford Royal Hospital), Yik Roy Hwang (Darlington Memorial Hospital), Khadija Khan (Gateshead Health NHS Foundation Trust), Reeves Campbell (Newcastle Upon Tyne Hospitals NHS Foundation Trust), Rhea Suribhatla (John Radcliffe Hospital), charlotte johnson (Derriford Hospital), Archchun Karunananthan (Derriford Hospital), Nujha Begum (Torbay And South Devon NHS Trust), Cody Breese (Scunthorpe General Hospital), Ahmad Gulzar (Barnsley Hospital NHS Foundation Trust), Emma Nofal (Rotherham District General Hospital), Rina George (Doncaster Royal Infirmary), Kiran Stowell (Dorset County Hospital), Kiran Stowell (Royal Hampshire County Hospital), Alex Glendenning (Morriston Hospital Swansea), Kofi Cox (St George's Hospital), Panagiotis Kapsampelis (Kingston), Peter Crabtree (University College London Hospital), Angus Gao (Royal Free Hospital), Neal Patel (The Whittington Hospital), Shern Wai Koh (North Middlesex University Hospital), James Hernon (Norfolk And Norwich University Hospital), Giuseppe Preziosi (Queen Elizabeth The Queen Mother Hospital Margate), Hermes Manos (Victoria Hospital Kirkcaldy)

**United States**: Eli Adams (OSF Saint Francis Medical Center).

**Yemen Rep**.: Mohammed Al-Shehari (Al-Thawra Modern General Hospital).

**COLLABORATORS AND SUPERVISING CONSULTANTS**

**Algeria**: Benali Tabeti, Aicha Bengueddach, Anouar Remini, Hakima Kehili, Nabil Boudjenan Serradj, Nacim Ikhlef, Noureddine Chadeli, Hakim Larbi, Abdelkader Menasria, Chakib Behilil, Mohammed Elamine Meghaizerou (Ehu-1st November 1954).

**Australia**: David Proud, Nastassia Shulman, Wael Jamel, Hwa Ian Ong, Anh Vu, Felix Wang (Austin Hospital); Stephen Smith, Luke Peters, Saksham Gupta, Shane Zhang, Isaac Caitens, Colin House, Thomas Capomolla, You Jin Han, Timothy Walker, Peter Martin (Calvary Mater Newcastle); Kelvin Kwok, Mitchell Gooch, Shirley Cai, Jonathan Tandjung, Vanessa Ng, Joyce Wang, Emily Reid, James Cui, Dennis Shen, Andrew Li, Shawn Ng, Angeline Sathiakumar, Amanda Dawson, Jess Barklimore, Benjamin Julien, Gemma Qian, Janae Chew, Riya Bhatia, Jenny Siu, Nicole Li, Chakraborty Oung, Peter Hamer, William Lai, Sarah Maguire, Adi Naarayanan, Chi Wen Un (Gosford Hospital); Francesco Amico, Myrna Ishak, Linda Beukes, Aiden Cheung, Leannedra Kang, Auston Yu, Mina Al Tawel (John Hunter Hospital); Elizabeth Murphy, Yick Ho Lam, Carolyn Chew, Antonio Barbaro, Hong Lee, Charlotte Stennard, Angelyn Khong, Dylan Morley (Lyell McEwin Hospital); Matthias Wichmann, Alicia Lim, Ishraq Murshed, Mary Zhao, Stephanie Louey (Mount Gambier And Districts Health Service); Russell Hodgson, Zainab Naseem, Allen Xiao, Junghyun Nam, Shady Rizk, Kieran Benn, Emma Haege, Laura Bland, Mark Slavec, Kexin Sun, Yining Huang (Northern Hospital); Tarik Sammour, Luke Traeger, Hakim Fong, Yu Zhou, Jiangyu Zhou, Nicole Kate, Divyanshu Joshi (Royal Adelaide Hospital); Ruben Rajan, Alex Mimery, Talia Shepherd, Megan Banks, Tamika Bland, Bhargav Jayani, Conor Nash (Royal Perth Hospital); Abdallah Elsabagh, Pauline Miller, Joel Stein, Harshit Morisetty, Deanna Lee, Faisal Mohammed (St John Of God Midland Public And Private Hospital); Darren Tonkin, Dominic Parker, Qiwen Wang (The Queen Elizabeth Hospital); Richard G McGee, Shubhang Hariharan, Elizabeth Lun, Yannick De Silva, Betty Wang, Hugh Elbourne, Darvesh Singh Maan (Wyong Public Hospital).

**Bosnia And Herzegovina**: Samir Delibegovic, Mirhan Salibasic, Amela Komilija Efendic, Merima Kruscica, Emsad Halilovic (Clinical Center University Of Sarajevo).

**Bulgaria**: Dzhevdet Chakarov, Elena Hadzhieva (Umhat Sveti Georgi); Dobromir Dimitrov, Paulina Vladova, Meri Shoshkova, Martin Karamanliev, Aparajeya Shanker, Ananya Mehta, Mohamed Abdullahi, Vysakh Ratheesh, Vasan Kamalathevan, Anandu Sundar, Saheed Owolabi Shittu, Susanna Koshy Thomas, Chandini Vijay Kurup, Munazza Khan, Shashwat Shanker, Elahe Naghavi, Mitchelle Bernard D Silva, Christina Wiesner (University Hospital Dr Georgi Stranski).

**Colombia**: David Baquero, Juan Sebastian Lopez Figueroa, Daniela De La Ossa-Posada, Manuela Arbelaez-Gomez, Camilo Calderón Cano, Fernanda Villa Quijano, Juan Manuel Bermudez, Isabella Osorno Munoz, Manuela Restrepo Molina, Lorenzo Neira, Natalia Montes Suaza (Pablo Tobon Uribe Hospital).

**Croatia**: Dario Vukosav, Ivan Zekanovic, Ivanica Zupan, Jakov Mihanovic, Ivan Bacic (Zadar General Hospital).

**Egypt**: Mohamed Al Sayed, Mohamed Zidan, Hashem Altabbaa, Yousef Tanas, Youssef Kerolous, Mohamed Mokhtar, Youssef Okazy, Yasmine Seada, Marwan Bahnacy, Mostafa Seif El Deen, Abdelrahman Zidan, Albaraa Daradkeh, Osama Al Shaqran, Stephanie Hanna, Ahmed El Banna, Yassin Badr, Heidi Sherif, Abdalrhman Abousetta, Yasmena Gaber, Mohamed Mahmoud, Youmna Abourady, Mohamed Basha, Tarek Zaho (Alexandria Main University Hospital); Mohammed F. Ramadan, Mohamed Ibrahim, Abdallah Hussein, Sherif Alaa, Ahmed M. Kedwany, Eman Mohamed, Sarah Khaled, Esraa Kotb, Alshymaa Ebrahim, Samah Arafa, Moaiad Eldin A. Mohamed, Afnan Morad, Fatma Monib, Randa Soliman, Moamen Shalkamy, Mariam Nageh, Shehab Eldin Saad, Sohayla Youssef, Mohamed Shazly, Sahar Hassan, Mohamed Ragab, Asmaa Sedeek, Omar Arafat Mahmoud, Omar Salah, Abdelrahman A. Abdelrahman, Shahy Wael, Mariam Rayan, Mohamed Tarek Mahmoud, Amal Refaie, Gehad Kamel (Assiut University Hospital); Mohie El-Din Mostafa Madany, Sarah Abdelmohsen (Aswan University Hospital); Mohammed Bedair, Dina Hamed, Ahmed Saber Mohamed Abdelrahman (Giza International Hospital); Ahmed Abdallah, Abdullah N Nassar, Medhat Katry (Oncology Center Mansoura University); Moatassem Erfan, Mennatallah Elnokity, Shimaa Atiya, Mahmoud Reda, Dunia Mowafy (The Memorial Soaad Kafafi University Hospital).

**Germany**: Daniel Reim, Marie Christin Weber, Maximilian Berlet, Maximilian Kiessler (Technical University of Munich, School of Medicine and Health, TUM University Hospital, Department of Surgery); Tim Vilz, Willis Maria, Jan Gortzen Patin, Martin Sohle, Florian Recker, Maria Wittmann, Achilles Delis, Markus Velten, Mumtaz Koksal, Max Oremek, Linda Gosejacob, Niko Knulle, Nils Sommer, Elena Aleksandrova, Mike Stanke, Sven Klaschik, Philippe Kruse, Christian Bode, Pascal Kowark, Ana Kowark, Mark Coburn, Daniel Beel, Jonas Dohmen, Maximilian Oremek, Leona Baier, Andrea Kunsorg, Claudia Neumann, Tobias Hilbert, Nadine Strassberger Nerschbach, Ricarda Neubauer, Nicolas Borter, Dorothea Protte (University Hospital Bonn); Jorg Kleeff, Johannes Klose, Onur Bayram (University Hospital Halle).

**Greece**: Georgios Kavalieratos, Aikaterini Sarafi (Agios Savvas Anticancer Hospital); Ioannis Tierris, Christos Chouliaras (Athens Medical Center); Dimitrios Korkolis, Konstantinos Fousekis, Nikolaos Tasis, Dimitrios Manatakis (Athens Naval And Veterans Hospital); Nikolaos V Michalopoulos, Maria Papadoliopoulou, Angelos I Nikolaou, Polyxeni Alexiou, Efthymios Poulios, Spyridon Christodoulou, Panagiotis Kokoropoulos, Ioannis Hatzaras, Nikolaos Danias, Panteleimon Vassiliu, Nikolaos Arkadopoulos, Theodoros Sidiropoulos, Maria Papadoliopoulou, Ioannis Margaris, Dimitrios Sampanis (Attikon University General Hospital); Stylianos Kapiris, Aikaterini Paraskeva, Michail Psarologos, Aikaterini Paraskeva (Evaggelismos General Hospital); Georgios Kapogiannatos, Aristotelis Nikitaras, John Katogiritis, Prokopis Christodoulou, Christos Ioannides, Ioanna Gogoulou (General Hospital Asklepieio Voulas); Konstantinos Bouchagier, Georgios Ioannis Verras, Levan Tchabashvili, Francesk Mulita (General University Hospital Of Patras); Savvas Symeonidis, Elissavet Anestiadou, Konstantinos Zapsalis, Nikolaos Ouzounidis, Stefanos Bitsianis, Lydia Loutzidou, Vasileios Foutsitzis, Antonia Aikaterini Bourtzinakou, Konstantinos Siozos (George Papanikolaou General Hospital Of Thessaloniki); Konstantinos Toutouzas, Nikolaos Intzes, Anna Mexi (Hippocratio General Hospital); Stylianos Kykalos, Panagiotis Dorovinis, Myrto D Keramida, Athanasios Syllaios, Adam Mylonakis, Markos Despotidis, Fotios Stavratis, Annita Loizou, Konstantinos S Giannakopoulos, Michail Vailas, Aikaterini Mastoraki, Islam Kourampi, Ilias Vagios, Aouatif Erasmia El Kanty, Emmanouil Mylonakis, Dimitrios Schizas (Laiko University Hospital); Elissaios Kontis, Eleni Papamattheou, Lykourgos Katsiaras, Andreas Efstathiou (Metaxa Cancer Hospital); Eleftherios Spartalis, Georgia Schismenou, Michael Spartalis (Sotiria General Hospital Of Thoracic Diseases); Konstantinos Lasithiotakis, Georgia Petra, Ioannis Tsikritzakis, Eftychia Nikolaou (University Hospital Of Heraklion Crete).

**India**: Yashwant Sakaray, Kanai Debnath, Satyam Khanna, Irrinki Santosh, Satyajit Sarangi, Varsha Khandelwal, Satish S N, Anmol Singh, Anand Kothari (Post Graduate Institute of Medical Education and Research).

**Iraq**: Moayad Al Nakeeb, Sahar Mezher, Mustafa Joudah, Ahmed Fakeri, Karrar Al Mosawi, Abdulla Abdulla, Hayder Jabbar, Ahmed Al Janabi (Al-hussien Medical City).

**Ireland**: Helen Heneghan, Tom Gallagher, Robert O’Connell, Kealan Blake, Conor Gleeson, James O’Grady, Kate Eustace, Lily Farrell, Solveig Svendsen (St Vincent's University Hospital).

**Italy**: Stefania Cimbanassi, Maria Danieli, Michele Altomare, Andrea Spota, Roberto Bini, Giuliano Santolamazza, Martina Aguzzi, Francesca Nava, Francesco Virdis, Federica Renzi, Osvaldo Chiara, Margherita Carbonaro, Pietro Calcagno, Pietro Lombardi, Pietro Achilli, Lorenzo Morini, Bruno Alampi, Giovanni Ferrari, Vincenzo Nicastro, Irene Giusti, Pietro Carnevali (Asst Grande Ospedale Metropolitano Niguarda); Francesco Pata, Mariasara Osso (Azienda Ospedaliera Di Cosenza); Fabrizio Vittadello, Chiara Girotto, Andrea Grego, Enzo Mammano, Emanuela Tessari, Antonio Rella, Alvise Frasson, Luca Faccio, Giacomo Sarzo, Caterina Barrella, Emanuela Tessari (Azienda Ospedaliera Di Padova); Corrado Pedrazzani, Sabrina Zambelli Sopalu, Gabriele Gecchele, Angelo Di Vittori, Noemi Bicelli, Ernesto De Giulio, Riccardo Giuri, Giacomo Faccioli (Azienda Ospedaliera Universitaria Integrata Di Verona); Danilo Centonze, Costanza Distefano, Roberta Granata, Giordana Riccioli, Ludovica Stella, Rossella Gioco, Salvatore Costa, Domenico Zerbo, Massimiliano Veroux, Alessio Licciardello (Azienda Ospedaliero- Universitaria Policlinico San Marco); Adolfo Pisanu, Valentina Murzi, Tiziana Pilia, Federica Campus, Carla Piras, Piergiorgio Serra, Alessandra Saba, Mauro Podda, Emanuela Gessa, Paola Marongiu, Alessandro Cois, Federico Corronca, Silvia Montisci, Eleonora Locci, Marcello Pisano, Eleonora Silanos, Alessandro Carta (Cagliari University Hospital); Paola Germani, Sara Pepe, Davide Drigo, Letizia Cecchini (Cattinara University Hospital); Mauro Santarelli, Enrico Potenza, Beatrice De Zolt Ponte, Elena Montanari, Victor Ugo De Donato, Lorenzo Capello, Sara Galati, Micol Giuliano, Diego Visconti, Luca Benedetto Lo Piccolo, Sofia Gamba, Chiara Celano (A.O.U. Città della Salute e della Scienza di Torino); Domenico Soriero, Giacomo Carganico, Davide Pertile (Irccs Ospedale Policlinico San Martino); Paolo Delrio, Teresa Pagano, Daniele Sannino, Carmela Cervone, Alessia Aversano (Istituto Nazionale Tumori Fondazione); Enrico Restini, Ivana Conversano, Rocco Tumolo, Marco Varesano, Grazisa Scialandrone (Lorenzo Bonomo); Salomone Di Saverio, Marziali Irene, Martina Zambon, Grazia Travaglini, Laura Lely, Alberto Buonanno (Madonna Del Soccorso Hospital); Isabella Mondi, Eleonora Ciccioli, Federico Cavallari, Andrea Lana, Fjorenta Sulo, Mario Biral, Rolando Tasinato, Sebastiano Pillirone, Daunia Verdi (Mirano Hospital); Giorgio Ercolani, Francesca Tauceri, Leonardo Solaini, Daniela Di Pietrantonio, Massimo Framarini, Chiara Casadei (Morgagni-Pierantoni Hospital AUSL Romagna); Elise Pouli, Luca Schiavone, Alessandro Broglio, Martina Martorana (Ospedale Civile Di Voghera); Francesco Roscio, Anna Laureys, Tea Bergenti (Ospedale Di Circolo Di Busto Arsizio); Gianandrea Baldazzi, Marta Spalluto, Massimiliano Ardu, Giovanni Tarchi, Camillo Franzetti (Ospedale Di Legnano); Lorenzo Maria Fatucchi, Osvaldo Carpineto Savorani, Leonelle Lore Nguefack Noudem (Ospedale San Donato Usl Toscana Sud Est); Lorenzo Ripamonti, Giulia De Carlo, Veronica Brocco, Elenasofia Signaroli, Andrea Scacchi (IRCCS San Gerardo dei Tintori - Monza); Emanuele Romairone, Sara Marzorati, Lorenzo Epis, Salvatore Carrabetta, Francesco Floris, Andrea Pierre Luzzi, Ugo Giuseppe Ribeca, Raquel Diaz, Alice Filippelli, Salvatore Carrabetta, Dorena Caruso, Fabrizio Ballari, Carolina Righetti, Pietro Grondona, Francesca Re (Ospedale Villa Scassi); Vincenzo Lizzi, Marco Montagna, Alessandra Giuliani, Francesco Maffei, Rocco Melino, Nicola Tartaglia, Fernanda Vovola, Giovanna Pavone, Mario Pacilli, Alberto Gerundo, Giovanni Di Gioia (Ospedali Riuniti Azienda Ospedaliera Universitaria Foggia); Andrea Mingoli, Gioia Brachini, Bruno Cirillo (Policlinico Umberto I); Eugenio Cucinotta, Santino A Biondo, Teresa Sinicropi, Francesco Fleres, Giuseppe Martorana, Vincenzo F Tripodi, Carmelo Mazzeo (University of Messina, Messina); Alessandro Spaziani, Emanuela Basile, Gaetano Poillucci (San Matteo Degli Infermi); Martina De Leonardis, Irene Tucceri Cimini, Danilo Meloni, Antonella Grasso (San Salvatore Hospital, University of L’Aquila); Maurizio Cervellera, Lodovico Sartarelli, Claudia Mongelli, Francesco Bagnardi (Santa Annunziata Hospital); Giovanni Terrosu, Lara Bonello, Davide Muschitiello, Vittoria Morinelli, Federica Passafiume (Ospedale Santa Maria della Misericordia di Udine); Antonello Deserra, Alessandro Cannavera, Francesca D Agostino, Carla Margiani, Cristina Murru, Giada Pattaro, Barbara Demurtas (Santissima Trinità - Ats Sardegna); Giuseppe Ietto, Simone Gianazza, Elisa Monti, Giacomo Borroni, Stefano Megna, Eugenio Cocozza, Lorenzo Livraghi, Roberto Delpini, Mattia Berselli, Davide Inversini, Enrico Ferri, Andrea Palillo, Domenico Iovino, Sabrina Garbarino, Alessandra Zullo, Chiara Peverelli, Valeria Quintodei, Valentina Marchionini, Lorenzo Conti, Alessandro Marzorati, Elisabetta Marta Colombo, Murad Odeh, Giuglio Carcano, David Meierruth (University Of Insubria)

**Jordan**: Mohammad Salah, Mus Ab Elatrash, Faris Abbadi (Al-Tafilah New Hospital); Motasem Almaletti, Yanof Al Naggar, Mohammad Salah, Sief Addeen Al Tahayneh (Islamic Hospital); Mohammad Sami Elmuhtaseb, Osama Abdul Kareem Sarhan, Almu’atasim Khamees, Raghad Yousef Yassin, Khalifa Salem Augi, Noorelhuda Kamal Abubaker, Seba Mahmoud Alghananeem, Ayat Khaled Mohsen, Aseel Ahmad Rezeq (Jordan University Hospital); Khaled Obeidat, Mahmoud Mahafdah, Saleh Shammakh, Hasn Haj Freej, Suleiman Mahafdah (King Abdullah University Hospital/ Jordan University Of Science And Technology); Anas Aljaiuossi, Mohammad Tanashat, Obieda Altobaishat, Samah Alananzeh (Princess Basma Hospital).

**Latvia**: Kristine Pavlovica, Daniella Zvina, Anzelika Beikule, Hoshika Tharni Sivapalan, Aija Tumova, Ilvija Knospina, Alise Antuanete Snikere, Anna Borisova, Sibi Anpalagan, Luxsana Mathiyalagan, Zhizi Yang, Andrejs Tolstiks, Nityanand Jain, Renate Ruta Apse, Asnate Matroze, Emija Nikola Karele, Sarmishtha Sharma, Deepkanwar Singh Panag, Aybaniz Ahmadova, Rohela Amiry, Olegs Zasibajevs (Pauls Stradins Clinical University Hospital).

**Libya**: Mostafa Elawami, Naseralla Elsaadi, Ebtisam Elbraky, Azza Eloshibi, Fayrouz Hweidy, Asma Moftah, Wijdan Sayfulnasr, Randa Ateh, Montaser Benzayed (Benghazi Medical Center); Hamida El Magrahi, Abir Ben Ashur, Salem Ali (Crown Health Care Clinical Team); Hussain Aboudlal, Akram Alkaseek, Haitam Shames, Anas Salim Abd Zayed, Amyirah Alshiteewi (Gharyan Central Hospital); Yamen Bobaker, Fatama Salem, Najway Tahir, Fatama Salem (Tobruk Medical Center); Sharfeddin Barka, Reem Ghmagh, Entisar Alshareea, Eman Abdulwahed (Tripoli Central Hospital); Ali Amer Ghummied, Suhaylah Timmalah, Serien Hossain Bensalem, Duaa Eisa Omar, Mohamed Alsori, Amira Ali Ashour, Rawia Jamal Hafed Ghmagh, Sara Mohammed Ammar Alsagheer (Tripoli Medical Center/ Tripoli University Hospital); Majdolin Miloud Almahjoub, Mustafa Ahmed Ekhail, Hajir Salem (Zliten Teaching Hospital)

**Lithuania**: Tomas Poskus, Marius Kryzauskas, Matas Jakubauskas, Kristina Marcinkeviciute, Ugne Silinskaite, Augustas Poskus (Vilnius University Hospital Santaros Klinikos).

**Malaysia**: Zaidi Zakaria, Michael Pak Kai Wong, Maya Mazuwin Yahya, Mohd Nizam Md Hashim, Wan Mokhzani Wan Mokhter, Wan Zainira Wan Zain, Siti Rahmah Hashim Isa Merican, Rosnelifaizur Ramely, Ikhwan Sani Mohamad, Syed Hassan Syed Abd Aziz, Jien Yen Soh, Mohd Azem Fathi Mohammad Azmi (School of Medical Sciences & Universiti Sains Malaysia Specialist Hospital); Tak Loon Khong, Sui Weng Wong, Mohamed Rezal Abdul Aziz, Amanda Weng Yee Leong, Wei Yang Heng (University Malaya Medical Centre).

**Mexico**: Mario Trejo Avila, Asya Zubillaga Mares, Daniel Astorga Guardado, Alejandra Nunez Venzor, Minnet Serrano Sanchez, Javier Andres Meza Hernandez (Hospital General Dr. Manuel Gea González); Noel Salgado Nesme, Oscar Santes Jasso, Danilo Tueme de la Peña, Oscar Posadas Trujillo, Daniel Doniz Gomez Llanos, Emilio Sanchez Garcia Ramos, Alberto Najera Saldana, Florencia Lucero Serrano, Jorge Canto Losa, Luis Davila Sanchez (Instituto Nacional De Ciencias Médicas Y Nutrición 'Salvador Zubirán').

**Morocco:** Elmehdi Boutajanouit, Youness Belattar, Hind Essalim, Ayman Echab, Mohamed Sami Melouane, Soulaimane Laaziri, Imane Oujaa, Ayoub Bousselham (Hopital Ibn Tofail); Amine Souadka, Mohammed Anass Majbar, Amine Benkabbou, Saloua Kassad, Arwa Aboumedian, Fatima Zahra Aboumedian, Othman Arsalan (Institut National D'oncologie).

**New Zealand**: Wal Baraza, Cameron Wells, Viha Vig, Genevieve Groult, Ashley Pereira, Josheel Ram, Hannah Stone, (Auckland City Hospital); Tamara Glyn, Mathew Morreau, Maia Wehi, Andreas Nicolaou, John Lu, Niamh Kilpatrick, Stella Daniell, Laura Sunderland, Zoe Williams, Analise Wang, Samuel Tomkins (Christchurch Hospital); Sze Lin Peng, Cameron Wells, Hannah Ashmore Price, Omar Nosseir, Marta Simonetti, Megan Singhal (Middlemore Hospital); Siraj Rajaratnam, Eileen Song, James Jin, Xiao Shen Hu, Caroline Stokowski, Anamitra Nair, Li Mei Gan, Vishvini Vijayakumaran, Si Jia Ong, Lisa Smith, Calvin Fraser, Varun Modi, Ethan Figgitt, Naeun Hwang (North Shore Hospital); Laura Brockie (Taranaki Base Hospital); Jeremy Rossaak, Binura Lekamalage, Jonathan Johns, Sophia Xu, Rebecca Veitch, Sunny Ding, Consuelo Alarcon, Yash Shahri, Claudia Thomas, Julia Shearer, Daniel Carson (Tauranga Hospital); Jesse Fischer, James Uivaa, Monique Mahadik, Simon Lai, Emily Joe, Shaamnil Prasad, Maitreyi Jain, Ajay Bansal, Olga Korduke (Waikato Hospital); Sarah Rennie, Anahera Herewini, Clare Mcinerny Heather, Greer Jackways, Christina Gordon (Wairarapa Hospital); Anthony Lin, Sue Ong, Jeffery Tan, Mohammed H Alsinan, Tharinya Gamalath, Annelies Bardoul, Mairarangi Haimona (Wellington Regional Hospital); Christopher Harmston, Matthew Mcguinness, Oscar De Langen, Andrew Mcintyre Robinson (Whangarei Hospital).

**Nigeria**: Garzali Ibrahim Umar, Mustapha Abdullahi Muhammad, Tijjani Nasir Nagwamutse, Khadija Abdullahi Ado, Rifkatu Nasiru Alhassan (Aminu Kano Teaching Hospital); Oludolapo Afuwape, Oyeyemi Dada, Vincent Osoka, Adigun Ishola, Nurudeen Akinbami (University College Hospital); Samuel Olatoke, Taofiq Raji, Olushola Fasiku, Olufemi Arinde, Olayide Agodirin, Solomon Irmiya, Olalekan Agede, Saburi Oyewale (University Of Ilorin Teaching Hospital).

**Pakistan**: Tabish Chawla, Annam Kafeel, Madeeha Ali, Izza Tahir, Muhammad Mushahid Hussain Rizvi, Alinah Qureshi, Aqsa Amjad, Mohammad Shahzaib Qadir, Muhammad Mobeen Shahid, Muhammad Tabish Nasim, Saniya Waseem (Aga Khan University).

**Peru**: Cesar Huaroto Landeo, Juan Carlos Luna Cydejko, Diego Chavez Hernandez (Clinica Internacional).

**Poland**: Jakub Migoń, Michał Bąk, Adrianna Pielech, Natalia Torz, Jan Nicikowski, Joanna Osmólska, Dawid Murawa (University Hospital of Karol Marcinkowski in Zielona Góra).

**Portugal**: Helder Alem, Catarina Santos, Nuno Mendonca, Patricia Bernardo, Sara Patrocinio, Frederico Nazareth, Lourenco Moniz (Centro Hospitalar Barreiro Montijo); Catia Ferreira, Goncalo Guidi, Carolina Marques, Clara Leal, Andre Silva, Margarida Dupont (Centro Hospitalar De Trás-Os-Montes E Alto Douro); Pedro Silva-Vaz, Manuel Rosete, Maria João Amaral, Catarina Lopes, Rita Andrade, Diogo Paula, Tiago Antunes, Ana Filipa Vilela, Cristina Camacho, Ana Ruivo, Ines Prior, Adriana Ferreira, Pedro Pinto, Mariana Lemos, Ana Claudia Raposo, Raquel Teixeira (Centro Hospitalar E Universitário De Coimbra - Hospital Geral); Luisa Frutuoso, Joana Antunes, Egon Rodrigues, Penelope Correia, Miguel Magalhaes, Luisa Magno, Pryangka Martins, Maria Iglesias (Centro Hospitalar Entre O Douro E Vouga); Pedro Febra, Elisabete Pacheco, Joana Domingues, Rita Galama, Ines Seixo, Raquel Lalanda (Centro Hospitalar Médio Tejo); Miguel Cunha, Edgar Amorim, Juan Rachadell, Sofia Pina, Ines Miguel, Beatriz Dias, Pedro Almeida, Barbara Santos, Catarina Santos (Centro Hospitalar Universitario Do Algarve - Unidade De Portimão); Rita Camarneiro, Leticia Heeren, Regina Silva, Andrea Abreu, Barbara Tinoco (Hospital Das Caldas Da Rainha - Centro Hospitalar Do Oeste); Olena Teslyak, Aldo Jarimba, Renato Barradas, Beatriz Louro, Rita Lourenco, Nadia Marcos (Hospital De Santarem); Manuel Damasio Cotovio, Arnaldo Machado, Martim Rente, Rita Lima, Maria Isabel Pereira, Ines Matias, Ma Madalena Siqueira, Joana Bolota, Sofia Leandro (Hospital Do Espirito Santo); Diogo Sousa, Jessica Ricardo, Marilia Ferreira, Diana Stoian (Hospital Do Litoral Alentejano); Sandra Carlos, Brigitta Cismasiu, Ana Lucia Barreira, Joao Vaz, Francisco Vara Luiz, Miguel Palas, Susana Henriques, Jose Miguel Carlos (Hospital Garcia De Orta); Catarina Quintela Silva, Catarina Mesquita Guimaraes, Lilian Farias, Ligia Freire (Unidade Local De Saude De Matosinhos - Hospital Pedro Hispano).

**Qatar**: Zia Aftab, Ali Toffaha, Mohamed Abunada, Ammar Aleter, Mohammad Al Yaseen, Sherif Ahmed, Amjad Parvaiz, Mohamed Kurer, Omer Al Yahri, Murad Alahmad, Mohamed Khalaf, Mahmood Al Dhaheri, Ayman Ahmed, Salwa Sidahmed, Mahwish Khawar, Maram Salah (Hamad General Hospital).

**Russian Federation**: Tatiana Khorobrykh, Albina Zubayraeva, Akmalbek Otabekov, Aleksandra Koziy, Polina Panova, Bogdan Semchenko, Anna Rebrova (Im Sechenov First Moscow State Medical University); Aleksandr Butyrskii, Ferat Suleymanov, Server Kareymanov (Municipal Emegency Hospital No.6); Andrei Bazaev, Armen Kokobelan, Iliya Zhukov, Andrei Malov (Privolzhsky Research Medical University).

**Slovenia**: Jan Grosek, Tajda Kosir Bozic, Jurij Ales Kosir, Ales Tomazic (University Medical Centre Ljubljana).

**South Africa**: Adam Boutall, Vuyolwethu Soldati, Alex Muturi, Simphiwe Gumede, Zubeir Salie, Shrikant Peters, Kathryn Nieuwenhuis, Mayilan Chetty, Deborah Obeng, Robyn Brown, Bruce Biccard, Margot Flint (Groote Schuur Hospital).

**Spain**: Izaskun Balciscueta, Carla Leal, Christian Esteo, Christian Esteo, Sara Garcia, Jorge Febre, Beatriz Cueno (Hospital Arnau De Vilanova); Jose Martin Arevalo, Ana Izquierdo Moreno, Sara Palomares Casasus, Luisa Garzon Hernandez, Pablo Moya Marcos, Alba Perez Del Pozo, David Moro Valdezate (Hospital Clínico Universitario De Valencia); Carlos Guijarro Moreno, Ana Sanchez Gollarte, Enrique Gonzalez Gonzalez, Armando Galvan Perez (Hospital Del Henares); Luis Miguel Jimenez Gomez, Silvia Perez Ajates, Maria Sanchez Rodriguez, Monica Ballon Bordo, Alvaro Landeras Lopez, Carlos Morales Garcia, Daniel Velayos Herraez, Jose David Gonzalez Esteban, Cristina Rey Valcarcel, Monica Ballon Bordo, Jose David Gonzalez Esteban (Hospital General Universitario Gregorio Marañón); Sebastian Fernandez Arias, Leire Garcia Alonso, Pablo Del Val Ruiz, Ainoa Fraile Gonzalez (Hospital Universitario Central De Asturias (Huca)); Cristina Vera Mansilla, Manuel Diez Alonso, Belen Matias Garcia, Fernando Mendoza Moreno, Alma Blazquez Martin, Ana Quiroga Valcarcel, Enrique Ovejero Merino, Lucia Diego Garcia, Lucas Casalduero Garcia, Alberto Vilar Tabanera, Yousef Allaoua Moussaoui (Hospital Universitario Principe De Asturias); Jesus Abrisqueta, Angela Alcarazo, Noelia Ibanez (Hospital Universitario Virgen De La Arrixaca); Virginia Duran Munoz Cruzado, Carlos Javier Garcia Sanchez, Beatriz De Los Angeles Ruiz Garcia, Marta Garcia Corona, Paula Bravo Raton (Hospital Universitario Virgen Del Rocio); Hanna Cholewa, Vicent Primo, Blas Flor, Pablo Guerrero, Marta Nieto Sanchez, David Plazas, Alba Serrano, Jorge Sancho Muriel, Monica Millan, David Quevedo (Hospital Universitario Y Politécnico La Fe); Begona Estraviz Mateos, Manolo Leon Valarezo, Ana Uriguen Echeberria, Jaime Gonzalez Taranco, Laura Fernandez Gomezcruzado, Izaskun Markinez Gordobil (Hospital Urduliz); Maria De Los Angeles Mayo Ossorio, Ainhoa Maestu Fonseca, Alicia Inmaculada Alvarez Gonzalez, Ana Maria Camacho Oliva (Puerta del Mar University Hospital); Gerardo Chamoso Mialdea, Ricardo Jesus Castro Lara, Antonio Garcia Dominguez, Mercedes Estaire Gomez (Severo Ochoa University Hospital).

**Sri Lanka**: Malith Nandasena, Niruban Ganesarajah, Harikrishanth Shanmugaraj, Kalaiyukan Sathasivam, Dulanjana Ranasinghe, Minidu Chandraguptha, Hasangi Gamage, Shalika Nagasinghe, Jeewantha Senavirathna, Yohan Chamara, Kanchana Wijesinghe (Colombo South Teaching Hospital); Dakshitha Wickramasinghe, Deshan Gomez, Salma Salih, Duminda Subasinghe, Thamisha Nugaliyadda (National Hospital Of Sri Lanka).

**Sudan**: Mutasim Mursi Abubaker, Mojahid Hamdan Ahmed, Mohamed Musa Yassin, Ola Abdallah Eljizoly, Ahmed Mohamed Ibrahim Mohamed, Mohamed Ahmed Adam, Mohammed Salah Abduelrahman, Wtfaa Siddeg, Said Ibrahim (Gadarif Teaching Hospital); Elsamoul Abdulgafoor, Hayat Abuobaida, Essam Eldien Abuobaida (Ribat University Hospital).

**Switzerland**: Frederic Ris, Vaihere Delaune, Jeremy Meyer, Enrica Chiriatti, Isabelle Uhe, Abdul Ghyasi, Andrea Peloso, Emilie Liot, Guillaume Meurette (Geneva University Hospitals); Christos Andreou, Eliane Koller Brolese, Georgios Vergos, Joanna Naemi Marx, Lukas Eisner, Athanasios Tampakis (Kantonsspital Olten); Antonietta Petrusic, Popeskou Sotirios Georgios (Lugano Regional Hospital); Muhlhausser Julia, Stephanie Strauven, Anna Katharina Huber, Jorn Markus Gass (Luzerner Kantonsspital).

**Syrian Arab Republic**: Abdul Rahman Hammadieh, Gheed Abdul Khalek, Zienab Klib, Mohammad Rabee Mslmani, Kinan Nassar, Nafiza Martini, Majd Hanna (Al-Mouwasat University Hospital); Anwar Chammout, Abd Alwahab Alkhalaf, Shadi Haj Hussein, Hala Bakro, Ahmad Sajjee (Aleppo University Hospital).

**Turkey**: Afag Aghayeva, Gokalp Kagan Kurtoglu, Yilmaz Onat Koyluoglu, Metincan Erkaya, Esra Dogan, Mehmet Metin Yurttaser, Bengi Agca, Goktug Mert Kurtoglu, Abdurrahman Furkan Cetisli, Elvin Ay, Fran Ivan Cubranic, Sara Matulic, Emre Tunccan (Acibadem Altunizade Hospital); Tayfun Karahasanoglu, Melik Kagan Aktas, Bengu Togay, Ece Ada, Ilayda Esma Yavuz, Emre Tuzuner, Ismail Ahmet Bilgin, Tayfun Karahasanoglu, Mert Tanal, Bilgunay Ilkin Safa, Atahan Durbas, Yagmur Karatas, Bilgesu Duman, Furkan Demiral (Acibadem Maslak Hospital); Tayfun Bisgin, Muhammet Berkay Sakaoglu, Turugsan Safak, Mevlut Bugra Baysal, Beste Yildirim, Onur Karaagac (Dokuz Eylul Univ. Hospital); Osman Bozbiyik, Busra Kucukates, Sarp Tunali, Suleyman Unal, Ayse Tuzcuoglu, Eylul Turksever, Martin Andonov Mitkov, Kaan Okumus, Mert Anil Altun, Zeynep Selin Savas, Mustafa Ali Korkut, Ferda Ozkan, Tayfun Yoldas, Bahadir Emre Baki, Firat Basci, Zekeriya Erhan Akgun, Ilay Suleyman, Sonmez Sehim, Ece Irem Zaman, Batuhan Cakmak, Mehmet Baskayali, Berk Goktepe, Recep Temel, Gunay Huseynova, Ozge Kilinc, Sinem Kilicat, Sevgi Sena Demirci, Irem Guneri, Nilgun Izmirli, Rabia Sultan Atahan, Karya Islamoglu, Baris Ozkilic (Ege University Hospital); Ahmet Aslan, Sadik Kesmer, Banu Yigit, Serkan Yilmaz (Elazig Fethi Sekin City Hospital); Mustafa Oncel, Alparslan Saylar, Abdullah Emre Askin, Sebnem Bektas, Aylin Izgis, Mustafa Sefa Isin, Gozde Deniz, Meltem Yasar, Eyup Deniz (Istanbul Medipol University Hospital); Server Sezgin Uludag, Mehmet Faik Ozcelik, Haktan Ovul Bozkir, Berke Buyukarikan, Mehmet Erinc Onal, Didem Gulhan (Istanbul Universty - Cerrahpaşa Medical Faculty); Arif Atay, Hakan Kul, Mert Yoldas, Ozgem Uysal, Ali Alcan, Ayse Beyza Acik, Bilal Batuhan Yenen, Ezgi Su Albayrak, Furkan Baysal, Gulce Ozkayahan, Mehmet Oguz Pinar, Kaan Turmus, Mete Numan Etlik (Izmir Katip Celebi University Faculty of Medicine); Mustafa Cem Terzi, Semra Demirli Atici, Aras Emre Canda (Acibadem Izmir Kent Hospital); Mehmet Ulusahin, Adnan Gundogdu, Muhammet Ates, Hatice Bulut, Sule Sevim, Zeynep Kamak Surmen, Tayfun Surmen, Beyza Nur Ekmekci, Ayse Nilufer Yuzgec, Mustafa Deniz Tepe, Bilal Alkas, Merve Aktas, Ogun Bebek, Mahsima Gul Gumrukcu, Ece Karabulut, Mert Uzun, Mohammad Alnas Ah, Zeinab Danaei (Karadeniz Technical University Farabi Hospital); Emre Balik, Muhammed Ikbal Ates, Salih Nafiz Karahan, Mekselina Kalender, Furkan Camci, Arif Emir Narin, Dilara Yigit (Koç University Medical School); Tevfik Kivilcim Uprak, Ahmet Omak, Halil Ibrahim Sevindi, Sila Catal, Ozde Buda, Esin Zeynep Cinal, Burak Mert Saracoglu, Abbas Can Uludag, Asiye Sena Anli, Amer Salameh, Eminenur Sen, Muhammed Enes Tasci (Marmara University School of Medicine); Omer Faruk Ozkan, Elif Didem Terzi, Zafer Senol, Haron Cemel, Firat Demircan, Berkay Ozcan (Sultan 2. Abdülhamid Han Research and Training Hospital); Ibrahim Ethem Cakcak, Muhammed Samil Yekeler, Buse Ozkan, Selcen Demircioglu, Asli Kaya, Sarper Kizilkaya, Utku Yartasi, Eylul Senodeyici, Ilayda Bozkaya, Neslican Demir, Oya Budak, Arya Celikhasi, Mehmet Halil Keskin, Mutlu Can Pacaci, Kubra Nur Kabay, Mirac Ajredini, Merve Yaren Kayabas (Trakya University Hospital); Hamdi Ozsahin, Cenk Ersavas, Bulent Citgez (Uskudar University Faculty Of Medicine).

**United Kingdom**: George Ramsay, George Neelankavil Davis, Esyn Yeoh Ee Xin, Erica Ho Ching Tsoi, Prem Nagaraj, Stephanie Walker, Kirsty Luo-Yng Tay, Alishah Haider, Dominique Da Luz, Shubham Jain, Jasmine Luangboriboon, Anagha Chinmayee, Danielle Robertson, Tessa Yau, Miriam Lecerof, Savithri Sathivelu, Natthaya Eiamampai (Aberdeen Royal Infirmary); Michael Powar, Konstantinos Stasinos, Tanzil Rujeedawa, Hetta Friend, Jasmine Thomas, Wai Yan Ding, Lewis Witton, Fathima Manaal, Annie Zhao (Addenbrooke's Hospital); Colman Byrnes, Christopher Brown, Conor Mccollam (Antrim Area Hospital- Northern Health And Social Care Trust); Katharine Bevan, Abdul Hakeem, Seiver Karim, Jonathan Bennett, Zhenhao Lu, Arjun Sharma, Emily Smith, Iona Phillips, Ilias Epanomeritakis, Mathew Moolamannil, Anton Swarnan (Bedford Hospital); William Wallace, Aideen Campbell, Finn Mccann, Joyce Beshara, Catinca Ciuculete, Carlo Ferrazzano, Nial Connell, Sarah Mccandless (Belfast City Hospital); David Messenger, Shabnam Cyclewala, Matthew Kobetic, Alice Winch, Barbara Piecha, Tushar Rakhecha, Emma Baker, Stephen Downey (Bristol Royal Infirmary); Jamie Powell, Saidah Mohd Sahid, Chirag Goyal, Byapti Alice Nandi, Ethan Porritt, Chiara Dell Oro, Nikita James, Wafia Hussain, Ryhan Patel, Annie Alocious (Charing Cross Hospital); Natalia Sanchez Thompson, Sruthi Vatsavayi, Niharika Chokkapu (Chelsea And Westminster Hospital); Nicola Eardley, Aran Rees, Kaye Sparrow, Priyadarshini Padmanaban (Countess Of Chester Hospital); Manos Epanomeritakis, Jane Kilkenny, Donovan Campbell, Ronan Fegan, James Cartilage (Craigavon Area Hospital); Khalid Osman, Mazuin Abu Talib, Keith Mathew, Giustina Chu, Sulaymaan Al Majid (Darlington Memorial Hospital); Rina George, Jessica Banks, Freya Braddon (Doncaster Royal Infirmary); Hannah Easterbrook, Thomas Knowles, Cailin Ho, Zoe Austin (Dorset County Hospital); Panna Patel, Mohammed Kawsar, Ruiari Doherty, Zachary Campbell (Furness General Hospital); Thomas Hibbs, Paris Bruno, Mark Vipond, Fatima Sheerin, Euan Lewis, Eve Miller, Olivia Lark, Asha Jina, George Snell, Sajad Hussain, Anna Armstrong, Thomas Nicholls (Gloucestershire Royal Hospital); Deepa Bapu, Jessica Chang, Liam Phelan (Good Hope Hospital); Felicity Greenfield, Chris Thorn, Michael Okocha, Yegor Tryliskyy, Oceana Fernando, Alexander Kimish, Aminata Kaloko, Mya Patel Vathvali, Robert Seah, Farai Chiwah, Nirmitha Thayaparan, Sudais Naeem (Great Western Hospital); Alexis Schizas, Aliki Rompou, Kareem Omran, Leona Takeuchi, Hana Moattar, Ryan Sia, Duaa Faruqi, Jia Xin Khoo, Mosope Ajegbomogun, Nafisa Zilani, Miriam Frankl, Mahta Haghighat Ghahfarokhi, Hassan Kamal, Nadia Chowdhury (Guy's And St Thomas' Hospitals); Jalal Mohammad, Joseph Wyer (Heartlands Hospital); Vimal Hariharan, Dylan Whitaker, Shahin Zakeri, Jacinta Ngeh, Ehren Agarwal, Jianing You, Shiqing Ma, Katy Hempson (Hinchingbrooke Hospital); Sylvia Brown, Mishal Shahid, Nina Dworschak (Inverclyde Royal Hospital); Mark Bignell, Isobel Burridge, Shu Luk, Hanaa Asharaf, George Corby, Hanxiao Li, Hannah Nentwich, Imogen Wilkinson, Blessing Omorodion, Niamh Owens, William Thornton (John Radcliffe Hospital); Aarti Varma, Zoe Chia, James Bailey, Dylan Abeelack, Tanvi Mungale, Amrita Jandu, Arooj Qaiser, Rohit Ramesh, Rivya Mathews, Khafia Mehboob, Ashviny Ravindran, Ruta Dubinskaite (Lincoln County Hospital); Barrie Keeler, Ahmed Gendia, Dibyeshwari Rana, Aida Azlan, Lavanya Gupta, Raniah Al Saidi, Natasha Sheelam, Carlita Smith, Sonakshi Nemchand, Shreya Srikumar, Ragul Rajivan (Milton Keynes University Hospital); Dean Harris, Peter Cripps, Shwe Ooi, Samantha King, Lawrence Quach, Robyn Taylor, Colin Seel, Emma Harvey, Belinda Wang, Julia Bieniek, Bappy Basak (Morriston Hospital Swansea); Hema Sekhar, Mohammed Aradaib, Fouad Ashoush, Eleanor Kissane, Lucy Rimmer, Carol Koubaesh, Khan Mahrukh (Newcastle Upon Tyne Hospitals NHS Foundation Trust); Samer Zino, Ramy Shaalan, Vaishnevy Ganesh, Jenna Cook, Radhe Shantha Kumar, Sundas Butt, Rania Fernandes, Adibah Mohammad Amin, Joaquim De Sousa, Orla Busby, Tasnim Kouli, Sara Mohamed, Sameh Abdelwahab, Marcel Al Horoub, Shaimi Niraula, Sarah Virani, Ize Osagie (Ninewells Hospital); James Hernon, James Aldwinckle, Eunice Choi Yun Kwan, Dagmara Aulich, Francis Donya, Natalie Wheelhouse, Jungho Min, Sara Kazemzadeh, Mohammad Ali, Precious Ojo, Sho Giersztein (Norfolk And Norwich University Hospital); Lee Dvorkin, Ee Teng Goh, Michael Bath, Subramaniam Guru Naidu, Halil Hussein, Mamun Dornseifer, Niroshan Sivasothy, Lalana Songra, James Kersey, Tharindu Menushka Hansamal Galboda Liyanage, Shern Howe Koh (North Middlesex University Hospital); Elisabeth Drye, Mohamed Albendary, Abdulqudus Deeknah, Natasha Kowshik, Natasha Reid, Alwaleed Al Doory, Mikael Steede, Alan Hamda, Khai Saw (Peterborough City Hospital); Giuseppe Preziosi, Kulsum Maula, Nicole Claire Gentles, Aneeshka Nagpaul (Queen Elizabeth The Queen Mother Hospital Margate); Miss Catherine Boereboom, Hannah Boyd Carson, Francesca Ligori Malcom, Jahnavi Kalvala, Aashlesha Galla, India Jacklin Chatha, Manmeet Saundh, Titobiloluwa Coker, Ashrit Chohan, Bobbie Webster, Amelia Simenacz, Hawawu Muazu (Queens Medical Centre); Richard Slater, Alaa Obeida, Malaz Abbakar, Tariro Madziro, Hassan Farooq (Rotherham District General Hospital); Susan Moug, Hwei Jene Ng, Scott Macdonald, Aoife Carr, Emma Clark, Heather Craig, Rabia Ali, Mairi Cunningham, Mackenzie Green (Royal Alexandra Hospital); Nick Heywood, Alice Proctor, Sayed Mdabu, Khansa Irfan, Maneesha Weerasooriya, Amin Sohani, Joud Qasem, Shreya Sachdeva (Royal Blackburn Hospital); Nicholas Battersby, James Hughes, James Miller, Amy Stokes, Thibagaran Sathambihai, Dexter Sim, Alice Whitbread Abrutat (Royal Cornwall Hospital); Robert Bethune, Ben Rossi, Blazej Rybinski, Awais Chaudhary, Lavinia Cochetti, Sophie Thompson, Hannah Humphrey, Yara Lima De Mendonca, Pavel Loginovic, Eva Ruiz Daum, Burraq Imran (Royal Devon And Exeter Hospital); Jack Broadhurst, Kirsty Cole, Vinay Patel, Carlo Lori, Georgina Hart, Michelle John (Royal Hampshire County Hospital); Ondrej Ryska, Nader Helmy, Hamzah Amin, Elina Stokolova, Ferdos Rizgar, Saan Dyare, Fauzaan Syed, Shaheer Safdar (Royal Lancaster Infirmary); Mohamed Thaha, Theo Pelly, Yi Lun Khaw, Andreas Kakkou, Aleksandra Laguna, Jit Yih Tan, David Sinclair Thomas Junior, Anmol Kaur Dhaliwal, Navnit Kaur, Paraskevi Papatzanaki (Royal London Hospital); Alka Jadav, Edward Parkin, Ammarah Ughratdar, Bryant Chong, Namareq Ahmad, Maya Holt, Madeleine Truscott (Royal Preston Hospital); Muhammad Sajid, Abdul Malik Magsi, Pierre Jean Marie, Harveer Singh, Joey Fanstone, Keerthy Vijendran, Onyedi Moses, Courtney Ann Dennis, Reubeen Ahmad, Ioana Raducanu, Sana Jaan (Royal Sussex County Hospital); Isabella Sawyer, John Bunni, Bethany Wardle, Laura Gilliland, Florian Fischer, Isabelle Craner, Elise Bisson, Zara Coombes (Royal United Hospital Bath); Olivia Hurrell, Kathryn Mcwhirter, Shazna Bi, Ciara Mccaffrey, Christian Ward Bradley, Liza Buchynska (Royal Victoria Hospital); Jonathan Epstein, Emeka Nwokeocha, Oluwatobi Adegboye, Jeel Shukla, Imogen Phillips, Jude Geldart, Luqman Aizan, Hamza Shahbaz (Salford Royal Hospital); Rajeev Peravali, Alexia Farrugia (Sandwell General Hospital); Geeta Kaur, Sanjay Basu, Shaker Alseifi, Qaisar Razzaq, Ali Hamad, Michael Naguib, John Mcalister, Nicholas Dawson (Scunthorpe General Hospital); Paul Marriott, Zafar Shahbaz, Arun Osullivan, Harshita Buragapu, Abiramy Selvanathan, Lok Pong Cheng, Suki Bernstein (South Warwickshire NHS Foundation Trust); Cleo Kenington, Dimitra Peristeri, Kofi Cox, Zaynab Irfan, Sylvia Muthukkumaru, Isabelle Legood, Rishi Kumar, Raveena Khalsa, Joshua Asto, Charlotte Evans (St George's Hospital); Nasira Amtul, Candice Downey, Zaynab Hafeji, Elizabeth Burdekin, Sannah Jamil, Emily Armstrong, Bethany Bracewell, Alexander Randall, Liam Murphy, Daniel Kyeremateng, Lavesh Mirpuri, Shan Sunny, Lorna Blackmore, Sophie Price, Sagar Sanadi, Claire Hardy, Michaela Silver, Ruby O Loughlin, Benedict Mallucci, Rebecca Hakim (St James's University Hospital Leeds); James Kinross, Laura Tincknell, Yasmin Owadally, Pratik Ramkumar, Shneeza Gill, Dean Chughtai, Tianyu Song, Akshita Ramineni, Srikar Reddy Namireddy (St Mary's Hospital); Arnold Goede, Rachel Carten, Tomas Urbonas, Mariah Mwipatayi, Ameer Khamise, Santhosh Ayirookuzhi, Iona Banerjee, Beatrice Sorce, Anna Deal, Keya Agadi, Grace Scopes, Ali Waleed Khalid (Stoke Mandeville); Hasan Mukhtar, Omar Ugas, Harpreet Sekhon, Jessica Nyon, Aryan Goel, Drew Leamon, Harkirat Dhaliwal, Fope Adedeji, Zenel Qenami, Khadija Zribi, Juliet Kenstavica Pinto (The Whittington Hospital); Vidushi Sharma (Trinity College Dublin); Manish Chand, Tom Pampiglione, Taner Shakir, Hamza Mahmood, Adam Hussain, Lavine Liu, Sabrina Ali, Majlind Grozda, Rohma Shahzad, Vignesh Radhakrishnan, Hugo Ferreira, Aamina Mahmood, Mohammed Binyameen (University College London Hospital); Ghaleb Goussous, Vasileios Kalatzis, Sadhasivam Ramasamy, Emily Hall, Zaib Shamsi, Shiny Darwin, Tinashe Wadi, Julia Maciazek, Uzayr Sheikh, Ioannis Perros, Grace Bambury, Maanav Khanna (University Hospitals Of North Midlands); Michael Proctor, Alessandro Sgro, Julian Camilleri Brennan, Shannon Mcdonald, Jade Sheil, Rosie Young, Susan Chong, Susanne Marshall, Hermes Manos (Victoria Hospital Kirkcaldy); Jason Smith, Oliver Siaw, Zekiye Karagozlu, Akhil Sonecha, Ivin Jose, Haoze Yang, Ananya Jain, Andreas Zachariadis, Maryam Sheik, Hannah Hirji (West Middlesex University Hospital); Thomas Athisayaraj, Mojolaoluwa Olugbemi, Cheuk Man Lam, David Barnes, Lily Mainwaring, Aly Shaaban, Joanna Kucharczak (West Suffolk Hospital); Nicholas Ventham, Darren Porter, Emma Barron, Chantelle Sunley, Daniel Arbide, Reka Kovacs, Hariss G Paremes Sivam, Tze Yi Gan, Michelle Teo, Mathew Smith, Orieanna Reeve Chen (Western General Hospital); Conor Magee, Rebecca Reid, Farida Hegazy (Wirral University Teaching Hospital).

**United States**: Richard Anderson, Ayoolamide Gazal, Frank Disilvio, Eli Adams, Samantha Wahlers, Diego Ruiz-Avila, James Jackson, Devon Negron, Judy Suh, Kara Proctor (OSF Saint Francis Medical Center).

**Yemen Rep**.: Yasser Obadiel, Nashwan Tashan, Areej Alnajjar, Ragaa Albatool, Khloud Ali Saeed (Al-Thawra Modern General Hospital).

**DATA VALIDATORS**

**Algeria**: Anisse Tidjane (Ehu-1st November 1954).

**Australia**: Felix Wang (Austin Hospital); Hannah Legge Wilkinson (Calvary Mater Newcastle); Upuli Pahalawatta (Gosford Hospital); Ishraq Murshed (Mount Gambier And Districts Health Service); Yui Kaneko (Northern Hospital); Jessica Hanna (Royal Adelaide Hospital); Leesha Bryan (Royal Perth Hospital); Mohammad Faraz, Bethany Cooper (St John Of God Midland Public And Private Hospital); Denise Chia (Wyong Public Hospital).

**Bulgaria**: Mick Galasyuk (University Hospital Dr Georgi Stranski).

**Colombia**: Erika Gabriela Miranda (Pablo Tobon Uribe Hospital).

**Croatia**: Ivo Coza (Zadar General Hospital).

**Egypt**: Mohamed Al Sayed, Mohamed Zidan (Alexandria Main University Hospital); Sarah Abdelmohsen, Mohie Madany (Aswan University Hospital); Sarah Mansour (Giza International Hospital); Ahmed Wael (Oncology Center Mansoura University); Ammar Yasser, Mahmoud Reda, Dunia Mowafy (The Memorial Soaad Kafafi University Hospital).

**Germany**: Daniel Reim, Marie Christin Weber, Maximilian Kie Ler (Klinikum Rechts Der Isar Tum School Of Medicine); Rica Philippi, Kilian Schmidt, Vera Guttenthaler (University Hospital Bonn); Maria Schuler (University Hospital Halle).

**Greece**: Aris Plastiras, Theodoros Tsirlis, Dimitrios Korkolis (Agios Savvas Anticancer Hospital); Evangelos Fradelos (Athens Naval And Veterans Hospital); Pantelis Kalogerakos (Attikon University General Hospital); Elena Mavrodimitraki, Dimitrios Stergiou, Konstantinos Polyzois (Evaggelismos General Hospital); Sandra Maria Tsoti (General Hospital Asklepieio Voulas); Ioannis Maroulis, Ioannis Panagiotopoulos, Panagiotis Perdikaris (General University Hospital Of Patras); Odysseas Lomvardeas, Ourania Kerasidou (George Papanikolaou General Hospital Of Thessaloniki); Orsalia Toutouza (Hippocratio General Hospital); Dimitrios Schizas, Christos Doudakmanis, Panagiotis Sakarellos (Laiko University Hospital); Ioannis Katsaros (Metaxa Cancer Hospital); Michael Spartalis (Sotiria General Hospital Of Thoracic Diseases).

**India**: Anmol Singh, Anand Kothari, Satyajit Sarangi (Post Graduate Institute of Medical Education and Research).

**Iraq**: Mustafa Al Obaidi (Al-hussien Medical City).

**Ireland**: Kate O’Shea (St Vincent's University Hospital).

**Italy**: Stefano Piero Bernardo Cioffi (Asst Grande Ospedale Metropolitano Niguarda); Nicola Passuello, Fabrizio Vittadello, Andrea Grego (Azienda Ospedaliera Di Padova); Riccardo Giuri, Giacomo Faccioli (Azienda Ospedaliera Universitaria Integrata Di Verona); Luigi La Via, Massimiliano Sorbello, Francesco Zagari (Azienda Ospedaliero- Universitaria Policlinico San Marco); Mauro Podda (Cagliari University Hospital); Francesco Velluti (A.O.U. Città della Salute e della Scienza di Torino); Raffaele Vincenzo (Irccs Ospedale Policlinico San Martino); Vincenza Granata (Istituto Nazionale Tumori Fondazione); Domenico Gattulli (Lorenzo Bonomo); Corrado Da Lio (Mirano Hospital); Anton Mariani Ivanikhin (Ospedale Civile Di Voghera); Francesco Roscio (Ospedale Di Circolo Di Busto Arsizio); Lorenzo Maria Fatucchi (Ospedale San Donato Usl Toscana Sud Est); Marco Ceresoli (Ospedale San Gerardo); Andrea Pierre Luzzi, Salvatore Carrabetta, Francesco Floris (Ospedale Villa Scassi); Vincenzo Lizzi, Nicola Tartaglia, Giovanni Di Gioia (Ospedali Riuniti Azienda Ospedaliera Universitaria Foggia); Carmelo Mazzeo, Francesco Fleres (University of Messina, Messina); Juliana Shahu (Santa Annunziata Hospital); Nicola Cillara, Alessandro Cannavera, Francesca D Agostino (Santissima Trinità - Ats Sardegna)

**Jordan**: Bourhan Alrayes (Islamic Hospital); Ayah Al-Qasrawi (Jordan University Hospital); Suleiman Mahafdah (King Abdullah University Hospital/ Jordan University Of Science And Technology).

**Latvia**: Pavils Plume, Arturs Truskovs (Pauls Stradins Clinical University Hospital).

**Libya**: Ebtisam Elbraky, Montaser Benzayed, Mostafa Elawami (Benghazi Medical Center); Hibah Bileid Bakeer, Akram Alkaseek (Gharyan Central Hospital); Mohammed Aljali, Munyah Mohammed (Tobruk Medical Center); Eman Abdulwahed, Reem Ghmagh (Tripoli Central Hospital); Ayyah Abdulfatah Altahir, Asraa Ali Alboaishi (Tripoli Medical Center/ Tripoli University Hospital); Najat Ben Hasan (Zliten Teaching Hospital).

**Malaysia**: Jie Soang Ooi, Seng Yeong Gan, Edmund Choong Yew Hoe (Hospital Universiti Sains Malaysia); Nora Abdul Aziz (University Malaya Medical Centre).

**Mexico**: Daniel Lopez Zertuche, Isabel Serrano Trejo (Hospital General Dr. Manuel Gea González); Stefano Minutti Galeazzi, Paola Tellez Castillo (Instituto Nacional De Ciencias Médicas Y Nutrición 'Salvador Zubirán').

**Morocco**: Iltimass Gouazar (Hopital Ibn Tofail); Raouf Mohsine (Institut National D'oncologie).

**New Zealand**: Jennifer Dang, Abrar Siddiquee (Auckland City Hospital); William Ju (Christchurch Hospital); Nicole Falkner (Middlemore Hospital); Kunj Joshi, Xiao Shen Hu, Calvin Fraser, Caroline Stokowski(North Shore Hospital); Jasmine Seidelin (Tauranga Hospital); Christina Gordon, Sarah Rennie, Anahera Herewini (Wairarapa Hospital); Tom Healy (Wellington Regional Hospital); Luke Paterson (Whangarei Hospital).

**Nigeria**: Fauziyya Tahir, Mustapha Ibrahim Usman (Aminu Kano Teaching Hospital); Peter Adeoye (University Of Ilorin Teaching Hospital).

**Pakistan**: Asad Saulat Fatimi (Aga Khan University).

**Poland**: Hubert Ficner, Wiktor Tworkowski (University Hospital of Karol Marcinkowski in Zielona Góra).

**Portugal**: Daniela Martins, Catia Ferreira, Goncalo Guidi (Centro Hospitalar De Trás-Os-Montes E Alto Douro); Pedro Silva-Vaz, Manuel Rosete, Cristina Camacho (Centro Hospitalar E Universitário De Coimbra - Hospital Geral); Luisa Frutuoso (Centro Hospitalar Entre O Douro E Vouga); Rita Galama (Centro Hospitalar Médio Tejo); Ana Rita Loureiro (Hospital Das Caldas Da Rainha - Centro Hospitalar Do Oeste); Helena Devesa (Hospital De Santarem); Diana Stoian (Hospital Do Litoral Alentejano); Madalena Trindade (Hospital Garcia De Orta); Ligia Freire (Unidade Local De Saude De Matosinhos - Hospital Pedro Hispano).

**Qatar**: Ibrahim Amer, Omar Moustafa, Noof Al Naimi (Hamad General Hospital).

**Russian Federation**: Aleksandr Butyrskii (Municipal Emegency Hospital No.6); Alexander Abelevich (Privolzhsky Research Medical University).

**Slovenia**: Jurij Ales Kosir, Tajda Kosir Bozic, Jan Grosek (University Medical Centre Ljubljana).

**South Africa**: Margot Flint, Simphiwe Gumede, Kathryn Nieuwenhuis (Groote Schuur Hospital).

**Spain**: Francisco Garcia Garcia (Hospital Clínico Universitario De Valencia); Ana Sanchez, Armando Galvan, Enrique Gonzalez (Hospital Del Henares); Beatriz Castro Catalan (Hospital General Universitario Gregorio Marañón); Fernando Mendoza Moreno (Hospital Universitario Principe De Asturias); Ana María Camacho Oliva, Alicia Inmaculada Álvarez González (Hospital Universitario Puerta del Mar); Noelia Ibanez, Angela Alcaraz, Jesus Abrisqueta (Hospital Universitario Virgen De La Arrixaca); Carlos Javier Garcia Sanchez (Hospital Universitario Virgen Del Rocio); Jorge Sancho Muriel, Hanna Cholewa, Monica Millan (Hospital Universitario Y Politécnico La Fe); Aitor Landaluce Olavarria (Hospital Urduliz); Maria Dolores Cancelas Felgueras, Elima Pilar Cagigal Ortega, Francisco Manuel Bujalance Cabrera (Severo Ochoa University Hospital).

**Sudan**: Mohamed Ahmed, Mohamed Salah, Mohamed Yassin (Gadarif Teaching Hospital); Essam Eldien Abuobaida (Ribat University Hospital).

**Switzerland**: Sofia El Hajji (Geneva University Hospitals); Lars Korber (Kantonsspital Olten); Jornj Markus Gass, Julia Muhlhausser, Stephanie Strauven (Luzerner Kantonsspital).

**Syrian Arab Republic**: Mohamad Klib (Al-Mouwasat University Hospital); Zahra Kasem (Aleppo University Hospital).

**Turkey**: Mert Tanal, Nur Ramoglu (Acibadem Maslak Hospital); Selvi Polat (Dokuz Eylul Univ. Hospital); Banu Yigit (Elazig Fethi Sekin City Hospital); Merve Aslan (Istanbul Medipol University Hospital); Ergin Erginoz, Server Sezgin Uludag, Mehmet Faik Ozcelik (Istanbul Universty - Cerrahpaşa Medical Faculty); Murat Aydemir (Izmir Katip Celebi University Faculty of Medicine); Aras Emre Canda, Mustafa Cem Terzi, Semra Demirli Atici (Izmir Kent Hospital); Hale Cepe (Karadeniz Technical University Farabi Hospital); Muhammed Ikbal Ates, Ibrahim Halil Ozata, Salih Nafiz Karahan, Derya Salim Uymaz, Serkan Sucu, Ahmet Rencuzogullari, Emre Balik, Dilara Yigit, Emre Ozoran, Mekselina Kalender, Arif Emir Narin (Koç University Medical School); Hasan Basri Yapici (Marmara University School of Medicine); Nurhilal Kiziltoprak, Elif Didem Terzi, Haron Cemel (Sultan 2. Abdülhamid Han Research and Training Hospital); Sila Nur Cansiz, Ahmet Hakan Nayman, Buse Selin Acar (Trakya University Hospital); Bulent Citgez (Uskudar University Faculty Of Medicine).

**United Kingdom**: Da Costa Dorkeh, Amira Orabi (Aberdeen Royal Infirmary); Jeffrey Tooze (Addenbrooke's Hospital); Lauren Mcgill, Matthew Killough (Antrim Area Hospital- Northern Health And Social Care Trust); Mathew Moolamannil (Bedford Hospital); Callum Auld (Belfast City Hospital); Thomas Sullivan (Bristol Royal Infirmary); Rahul Penumaka (Charing Cross Hospital); Kelly De Stadler (Chelsea And Westminster Hospital); Kathryn Allen (Craigavon Area Hospital); Richard Huynh (Darlington Memorial Hospital); Tim Wilson (Doncaster Royal Infirmary); Jadzia Chou (Furness General Hospital); Tanya Premi (Gloucestershire Royal Hospital); Rebecca Badminton (Great Western Hospital); Muhammad Aftab (Guy's And St Thomas' Hospitals); Shahin Zakeri (Hinchingbrooke Hospital); Vasu Sood (Inverclyde Royal Hospital); Nathan Appanna (John Radcliffe Hospital); Anas Saadeh (Lincoln County Hospital); Gopika Nair, Ragul Rajivan (Milton Keynes University Hospital); Eleanor Hopper (Newcastle Upon Tyne Hospitals NHS Foundation Trust); Caitlyn Gallagher, Vithya Vera (Ninewells Hospital); Lahreb Aktar (Norfolk And Norwich University Hospital); Haseeb Syed (North Middlesex University Hospital); Abdulqudus Deeknah (Peterborough City Hospital); Kirtana Ponnuswamy (Queen Elizabeth The Queen Mother Hospital Margate); Nuheel Iqbal (Queens Medical Centre); Alaa Obeida (Rotherham District General Hospital); Grace Lee, Yong Hong Jun, Hua Xuan Yeow (Royal Alexandra Hospital); Ghulam Majeed (Royal Blackburn Hospital); Thomas Smith, Khalid Bukhashem, Laura Munn (Royal Cornwall Hospital); Burraq Imran, Gauri Chillarge (Royal Devon And Exeter Hospital); Tricia Tay (Royal Lancaster Infirmary); Alexander Beneke (Royal London Hospital); Venkata Soumya Bodapati (Scunthorpe General Hospital); Vipanchi Katamaneni (South Warwickshire NHS Foundation Trust); Nika Majidi, Iman Anis (St George's Hospital); Hussain Hassan, Faizan Malik (St James's University Hospital Leeds); Puntrika Tannirandorn (St Mary's Hospital); Katharina Gollub (Stoke Mandeville); Timothy Grommet (University College London Hospital); Amarah Mirza (University Hospitals Of North Midlands); Alessandro Sgro (Victoria Hospital Kirkcaldy); Mahmoud El Khatib (West Middlesex University Hospital); Joanna Kucharczak (West Suffolk Hospital); Zhe Xuan Ho, Shazia Nusky (Western General Hospital).

**United States**: Matthew Grammer (OSF Saint Francis Medical Center)
